# A systematic characterization of fibroblast subtypes and heterogeneity

**DOI:** 10.1016/j.isci.2025.113915

**Published:** 2025-11-10

**Authors:** Xiuli Zhu, Hongen Kang, Zhongming Zhao, Peilin Jia

**Affiliations:** 1 China National Center for Bioinformation, Beijing, China; 2 Beijing Institute of Genomics, Chinese Academy of Sciences, Beijing, China; 3 University of Chinese Academy of Sciences, Beijing 100049, China; 4 Department of Biomedical Informatics, Vanderbilt University Medical Center, Nashville, TN, USA

**Keywords:** Technical aspects of cell biology, Cancer, Transcriptomics

## Abstract

Fibroblasts are essential components in development, disease, and treatment response, yet their diversity and function remain incompletely understood. Here, we constructed a comprehensive fibroblast landscape by integrating large-scale single-cell and single-nucleus RNA sequencing datasets spanning 60 major human organs and tissues across various developmental stages and biological conditions. We identified 16 fibroblast subtypes and systematically characterized their tissue specificities, transcriptional heterogeneity, and cell states. Further analyses revealed distinct fibroblast subtypes associated with pluripotency and senescence across tissues. Leveraging the well-defined fibroblast atlas, we demonstrated that different fibroblast subtypes were associated with various complex traits such as anthropometric and immune diseases. Furthermore, analysis using the subtype-specific gene activities in bulk cancer data revealed that myoCAF was a risk factor for patient survival. Collectively, our study provides a comprehensive fibroblast atlas, offering insights into the functions, heterogeneity, and clinical applications of fibroblast subtypes.

## Highlights

• Cross-tissue single-cell transcriptome analysis reveals fibroblast heterogeneity
• Niche-level cell-cell interactions between fibroblast subtypes and other cell types
• Fibroblast subtypes have unique pluripotency and cellular senescence features
• Fibroblast subtypes correlate with various complex traits and cancer types

## Graphical abstract

**Figure:**
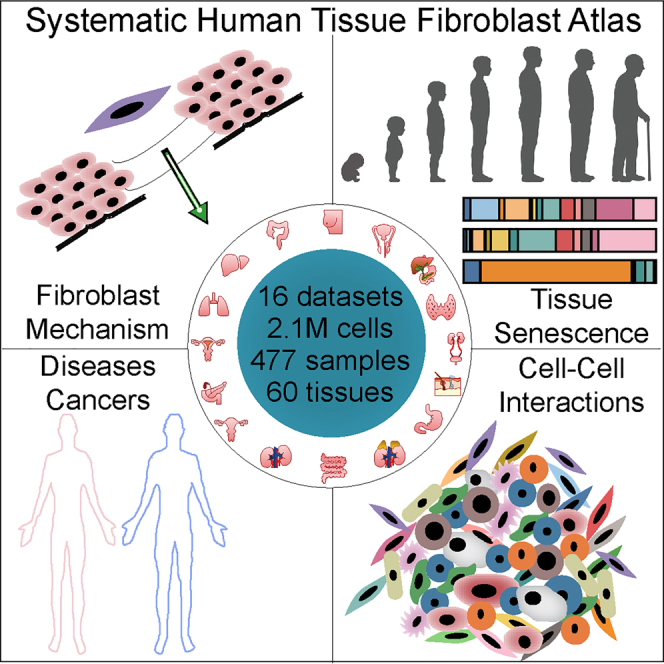

## Introduction

Fibroblasts are essential cells in maintaining the structural integrity and functions of various human tissues.^1^ They exhibit robust functional and metabolic capabilities, including protein synthesis and the maintenance of the extracellular matrix (ECM), enabling them to repair and remodel tissues in response to injury throughout growth and development. Fibroblasts are distinct from fibrocytes, where the latter are derived from circulating monocytes and play a role in tissue repair by differentiating into fibroblasts at the sites of injury. Thus, fibrocytes are considered a transitional state between circulating monocytes and active fibroblasts.^2^ In this work, we focused on fibroblasts, especially their subtypes, functional characteristics, and impacts on complex diseases, including cancer.

Dysfunctions of fibroblasts can contribute to the pathogenesis of complex disease.^3^ For example, in pulmonary fibrosis, aberrant activation and proliferation of lung fibroblasts generate excessive ECM deposition, leading to progressive lung scarring and impaired gas exchange.^4^^,^^5^ With conditions such as heart failure and myocardial infarction, dysregulated cardiac fibroblasts contribute to cardiac fibrosis, which impairs myocardial function and increases the risk of arrhythmias and heart failure progression.^6^^,^^7^ In diabetic nephropathy, dysfunctional renal fibroblasts are attributed to the development of renal fibrosis and progressive loss of kidney function.^8^^,^^9^ In cancer, cancer-associated fibroblasts (CAFs) represent abnormally activated ECM cells that emerge during the early stage of tumor development. CAFs secrete growth factors, cytokines, and ECM components that facilitate tumor cell proliferation, metastasis,^10^^,^^11^ and immune evasion.^12^

Fibroblasts can also be characterized based on their pathway activities, cellular states, responses to environmental stimuli, and interactions with other cell types. Transforming growth factor β signaling is a central pathway regulating fibroblast activation and ECM synthesis in fibrosis and cancer. Specific fibroblast states are increasingly recognized as regulators of immune cell function in inflammatory diseases and cancer.^13^ The emergence of senescent cells is generally associated with aging and the decline of tissue regeneration.^14^ Senescent fibroblasts exhibit altered gene expression profiles, secretion of pro-inflammatory cytokines and growth factors, and changes in cell morphology and function.^15^ Fibroblasts with cellular senescence properties are present in healthy epithelial tissues, supporting stem cell function and tissue repair.^16^ Fibroblasts can also undergo senescence in response to various stresses, such as oxidative stress, DNA damage, oncogene activation, or exposure to pro-inflammatory cytokines. Finally, crosstalk between fibroblasts and other cells can modulate fibroblast activation and tissue remodeling, playing a critical role in both healthy and diseased conditions.

Fibroblasts can be further divided into subtypes that exhibit functional and phenotypic heterogeneity.^17^^,^^18^ Depending on the location, normal dermal fibroblasts are divided into papillary fibroblasts (PapFBs) and reticular fibroblasts. PapFBs are located in the upper layer of the corium and are involved in the formation of skin hair follicles and immune responses. Reticular fibroblasts are located in the lower layer of the corium and are responsible for making skin collagen fibers that trigger the repair of damaged skin.^19^ The aforementioned CAFs are mainly derived from fibroblasts inherent in the interstitium surrounding the tumor and can also be formed by epithelial cells, endothelial cells, or mesenchymal stem cells through a series of signal transformations. At least three subtypes of CAFs have been reported: myofibroblastic CAF (myoCAF), inflammatory CAF (iCAF), and antigen presentation CAF (apCAF),^20^ each with distinct functions. Buechler^21^ identified the universal fibroblasts (UniFBs) in steady-state tissues and activated fibroblasts (ActFBs) in perturbed tissues. Gao^22^ and Liu^23^ reported 20 and 18 distinct fibroblast subtypes, respectively, classified based on highly expressed genes in each subtype. Nevertheless, the field still lacks a unified fibroblast classification system, as increasing numbers of studies continue to emerge using similar approaches but focusing on more limited disease- or organ-specific contexts. Consequently, the nomenclature for fibroblast phenotypes has become highly inconsistent, creating significant challenges when comparing fibroblast subsets across different datasets due to varying naming conventions.

Considering the diverse functions and dynamic states of fibroblasts, a systematic investigation is pressingly needed to delineate potential subtypes of fibroblasts and elucidate their tissue-specific functions in both health and disease. In this study, we conducted a pan-tissue investigation of the fibroblast subtypes using single-cell RNA sequencing (scRNA-seq) or single-nucleus RNA sequencing (snRNA-seq) data from representative human tissues and organs, aiming to construct the landscape for fibroblast subtypes. Through trajectory analyses, we unveiled the diverse directions of fibroblast differentiation and then cross-validated the results using multiple analytical approaches. To elucidate the unique characteristics of fibroblast subtypes, we examined the transcriptional heterogeneity among different subtypes, focusing on cell states, regulatory networks, and functional mechanisms. Furthermore, we investigated the influence of aging on these subtypes and uncovered distinct features related to cell pluripotency and cellular senescence. Finally, we demonstrated that such comprehensive annotation of fibroblast subtypes provides valuable insights into the cellular context of complex traits and diseases, including cancer.

## Results

### The landscape of fibroblasts across human tissues

To examine the distribution of fibroblast cells in various tissues and biological conditions, we constructed a comprehensive landscape including 2,195,794 cells from 16 scRNA-seq or snRNA-seq studies spanning 60 major human organs or tissues (supplemental information, Tables 1 and S1). These data were generated from 477 samples representing different biological conditions, including normal, disease, cancer, and abortion (Tables 1 and S1). We implemented a series of quality control and processing, including exclusion of low-quality datasets and cells, correction for potential batch effects, initial clustering and merging, and evaluation of the clusters using multiple parameters (e.g., Spearman correlation coefficient [SCC], dendrogram, and t-distributed stochastic neighbor embedding) (Figures S1–S8; Table S1. Clinical characteristics of collected samples, Table S2. Statistics about the 9 major cell types, Table S3. Statistics of the differentially expressed genes for each cell cluster, Table S4. The annotations of fibroblast subtypes, Table S5. The marker genes for fibroblast subtypes, Table S6. Statistics of fibroblast subtypes in collected cohorts; supplemental information).

**Table 1:** Statistics of the 16 datasets

Datasets	Tissues	#FB cells by metadata	#FB cells by harmony	#FB cells by NES	#FB cells by CellTypist	#FB cells after QC
GSE153643	Adipose & liver	4,596	6,111	6,106	5,442	5,441
GSE123813	Basal	378	1,375	1,375	595	595
GSE145137	Bladder	273	459	459	322	322
GSE132257	Colorectal	953	48	47	18	18
GSE144735	Colorectal	7,650	3,145	3,140	2,190	2,189
GSE160269	Esophageal	37,213	40,048	40,048	973	973
GSE109816	Heart	406	0	0	0	0
GSE121893	Heart	973	0	0	0	0
GSE130148	Lung	204	337	311	15	15
GSE131907	Lung	4,172	4,256	4,243	2,588	2,587
GSE158127	Lung	31,032	34,746	34,673	24,440	24,437
GSE171524^a^	Lung	21,472	22,302	20,647	9,780	9,263
GSE150430	Nasopharyngeal	0	31	31	18	18
GSE159929	15 Tissues	15,163	21,024	21,008	15,023	15,021
GSE201333	24 Tissues	40,870	77,111	55,643	59,635	46,515
GSE134355	60 Tissues	91,356	136,695	114,471	55,901	50,590
Total	60 Tissues	256,711	347,688	302,202	176,940	157,984

FB, fibroblast; NES, normalized enrichment score; QC, quality control.

a All datasets were generated using scRNA-seq, except that GSE171524 was generated using single-nucleus RNA sequencing.

**Figure 1:**
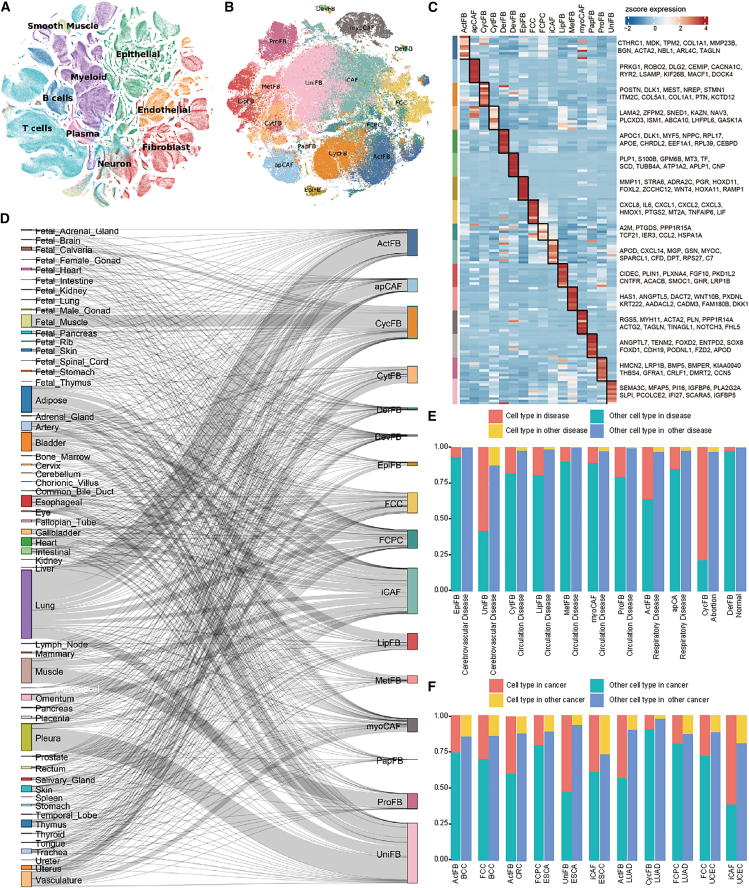
The landscape of fibroblasts across human tissues (A) The t-distributed stochastic neighbor embedding (t-SNE) plot of all cells colored by major cell types. (B) The t-SNE plot of the fibroblast cells colored by fibroblast subtypes. (C) Heatmap showing the average expression patterns of marker genes of 16 fibroblast subtypes. The left annotation bar represents corresponding fibroblast subtypes (expression profile in Table S5). (D) The Sankey chart of the fibroblast subtypes and tissue. (E and F) Bar plots showing whether fibroblast subtypes are significantly enriched in a specific disease (E) or cancer types (F), assessed by the chi-square test. The bar size represents the percentage of the specific fibroblast subtype. Red bar represents the percentage of the fibroblast subtype in a disease/cancer, cyan bar represents the percentage of other fibroblast subtypes in a disease/cancer, yellow bar represents the percentage of fibroblast subtype in other disease/cancer, and blue bar represents the percentage of other fibroblast subtypes in other disease/cancer.

As a result, we obtained 2,062,895 cells from 368 samples from 14 studies. These cells were categorized into 9 major cell types: myeloid cells, B cells, T cells, plasma cells, epithelial cells, endothelial cells, neurons, smooth muscle cells, and fibroblast cells (Figures 1A and S9A; Table S2). We next focused on the fibroblasts (Figures S4–S8; Table S3. Statistics of the differentially expressed genes for each cell cluster, Table S4. The annotations of fibroblast subtypes, Table S5. The marker genes for fibroblast subtypes, Table S6. Statistics of fibroblast subtypes in collected cohorts). To define a high-quality set of fibroblasts, we further filtered the fibroblasts by keeping only those supported by both our pipeline and the original metadata, resulting in 157,984 fibroblast cells for further analysis (45.4% of 347,688 fibroblasts from our pipeline, 55.9% of 256,711 originally annotated fibroblasts) (Figure S1; Table 1). This subset of high-quality fibroblasts was thoroughly validated using multiple measurements, such as well-known marker genes *COL1A1* and *COL1A2* (83.4% of cells had a count value >1 for *COL1A1* or *COL1A2*, Figure S4F). Further applying clustree^24^ with the Louvain algorithm, followed by assessment using the ratio of global unshifted entropy (ROGUE) algorithm,^25^ we identified 41 clusters, which were then merged into 16 fibroblast subtypes based on their shared differentially expressed genes (DEGs) (Table S3) and the average ROGUE values. Names of these subtypes were based on recommendations from CellMarker2^26^ or their tissue origins (Figures S6–S8; Tables S4 and S5).^26^ Moreover, we collected fibroblast-related marker genes of normal and tumor tissues from CellMarker2 and calculated a module score for the normal state and CAF-like state, respectively, for the 16 fibroblast subtypes using the AddModuleScore function in Seurat. For the normal state, we collected 331 marker genes, and for the tumor state, we collected 57 marker genes from the CellMarker2 database (Figure S8A). We then performed a paired two-sided *t* test on the module scores between the normal and CAF-like states for each subtype, followed by Benjamini-Hochberg-correction for multiple testing (adjusted *p* value <0.05) (Figure S8B). Strikingly, all subtypes showed a significant difference between the two states. According to the scale of the two states, we stratified the 16 fibroblast subtypes into two groups: the normal-like group (DevFB, DerFB, PapFB, EpiFB, LipFB, MetFB, ProFB, and CytFB) and the CAF-like group (myoCAF, apCAF, CycFB, FCC, UniFB, FCPC, iCAF, and ActFB). More details of the preprocessing steps can be found in the supplemental information.

These 16 subtypes are ActFB (*n* = 14,296 cells), apCAF (*n* = 7,012 cells), cycle fibroblast ([CycFB] *n* = 17,434 cells), cytoskeleton fibroblast ([CytFB] *n* = 9,088 cells), dermal fibroblast ([DerFB] *n* = 1,052 cells), developmental fibroblast (DevFB, *n* = 883 cells), epithelial-like fibroblast ([EpiFB] *n* = 2,001 cells), fibrocartilage chondrocyte cell (FCC, *n* = 10,909 cells), fibrochondrocyte progenitor cell (FCPC, *n* = 9,727 cells), iCAF (*n* = 24,574 cells), lipid fibroblast (LipFB, *n* = 8,691 cells), metabolic fibroblast (MetFB, *n* = 4,100 cells), myoCAF (*n* = 7,256 cells), PapFB (*n* = 182 cells), proliferation fibroblast (ProFB, *n* = 8,297 cells), and UniFB (*n* = 32,482 cells) (Figures 1B and 1C; Tables 1 and S6). We calculated the SCC of the 8 major cell types and 16 fibroblast types using their average expression. Using unsupervised hierarchical clustering, we observed two clusters, one including 16 fibroblast subtypes and the other including 8 major cell types (Figure S9A). The distance among fibroblast subtypes was closer (Figure S9B, median SCC among 16 fibroblast subtypes = 0.891, median SCC among 8 major cell types = 0.874, median SCC among 8 major cell types and 16 fibroblast subtypes = 0.786), indicating their similar biology characterizations (Figure 1B).

### Tissue-specific fibroblast subtypes

These 16 fibroblast subtypes were distributed across 56 tissues, with four tissues in the original datasets having no fibroblast cells, i.e., epityphlon, blood, fetal eyes, and fetal liver. The fibroblast cells exhibited high heterogeneity across tissues (Figure S9E). Of note, fetal and adult tissues each included distinct fibroblast subtypes. For example, UniFB was widely present in nearly all non-abortion tissues, indicating its ubiquitous roles.^21^ In contrast, CycFB was prevalent in various abortion tissues (Figure S10). apCAF, iCAF, and myoCAF were detected in most diseases and cancer tissues.^27^^,^^28^ DerFB, FCC, FCPC, LipFB, and PapFB exhibited tissue-specific distributions and have been reported in previous studies.^20^^,^^21^^,^^29^^,^^30^^,^^31^^,^^32^^,^^33^^,^^34^ Among the 16 identified fibroblast subtypes, 10 displayed concordances with previous subtypes, while 6 were putatively not reported, namely, CycFB, CytFB, DevFB, EpiFB, MetFB, and ProFB (Table S7). Due to limitations in experiment and lack of access to tissue specimens, we instead estimated the components of these 16 fibroblast subtypes, along with 8 other cell types in The Cancer Genome Atlas (TCGA) bulk RNA sequencing (RNA-seq) datasets by CIBERSORT (Table S8).^35^ The deconvolution results provided independent supporting evidence for the presence of these fibroblast subtypes (Figure S11).

The Sankey plot in Figure 1D displays the relationships of fibroblast subtypes, highlighting the strong heterogeneity in tissue development. Among all fibroblast cells, lung tissue constituted the predominant proportion (23.3%, *n* = 36,818 cells), followed by pleural tissue (9.2%, *n* = 14,559 cells) (Figures 1D and S9E). Notably, CycFB was significantly enriched in fetal tissues (Figure S10). The original dataset comprised four major categories: normal samples (13 tissues, 56 samples, 31,845 cells), disease samples (34 tissues, 125 samples, 101,030 cells), cancer specimens (13 cancer types, 89 samples, 8,385 cells), and abortion cases (18 tissues, 33 samples, 16,724 cells) (Figures S12A–S12C). Particularly for the disease samples, we annotated them into 8 groups following the International Classification of Disease-11 codes. Interestingly, our analysis revealed 9 fibroblast subtypes significantly over-represented across three disease groups (Figure 1E). Specifically, cerebrovascular diseases showed prominent enrichment of EpiFB and UniFB subtypes; respiratory diseases, including COVID-19, asthma, idiopathic pulmonary fibrosis, and respiratory failure, demonstrated significant over-representation of ActFB and apCAF subtypes; and circulatory system diseases (primarily anoxia and hypertension) displayed the broadest subtype spectrum with significant enrichment of CytFB, LipFB, MetFB, myoCAF, and ProFB. Furthermore, CycFB was mainly identified in abortion samples (chi-square test, *p* < 2.2 × 10^−16^), while DerFB was mainly identified in normal samples (chi-square test, *p* < 2.2 × 10^−16^).

Among the 13 cancer types, the distribution of fibroblast subtypes exhibited marked heterogeneity too (Figures 1F and S12D; Tables S9 and S10). We identified 11 significant associations between fibroblast subtypes and cancer types. Notably, the ActFB subtype was the most prevalent, showing significant enrichment in basal cell carcinoma (BCC; chi-square test, *p* value = 1.19 × 10^−13^), colorectal cancer (CRC; *p* value = 5.69 × 10^−128^), and lung adenocarcinoma (LUAD; *p* value = 2.56 × 10^−214^). Three subtypes were enriched in two cancer types each: FCC was enriched in both BCC (*p* value = 6.87 × 10^−27^) and uterine carcinosarcoma (UCEC; *p* value = 1.99 × 10^−275^), FCPC in esophageal carcinoma (*p* value = 6.87 × 10^−27^) and LUAD (*p* value 1.99 × 10^−275^), and iCAF in esophageal squamous cell carcinoma (*p* value = 5.33 × 10^−28^) and UCEC (*p* value = 8.77 × 10^−12^).

Among the cancer samples, 6 CRC patients were each sequenced at three sites: a core and a border sample from tumors and a normal mucosa sample. We thus examined fibroblast compositions across different sites to assess their spatial variations (Table S10). As shown in Figure S12E, several subtypes displayed substantial site-specific differences. For instance, UniFB and iCAF were primarily observed in normal mucosa, whereas ActFB and myoCAF were enriched in tumor-associated samples (core or border).

### Characteristics of fibroblast subtypes

#### Trajectory

**Figure 2:**
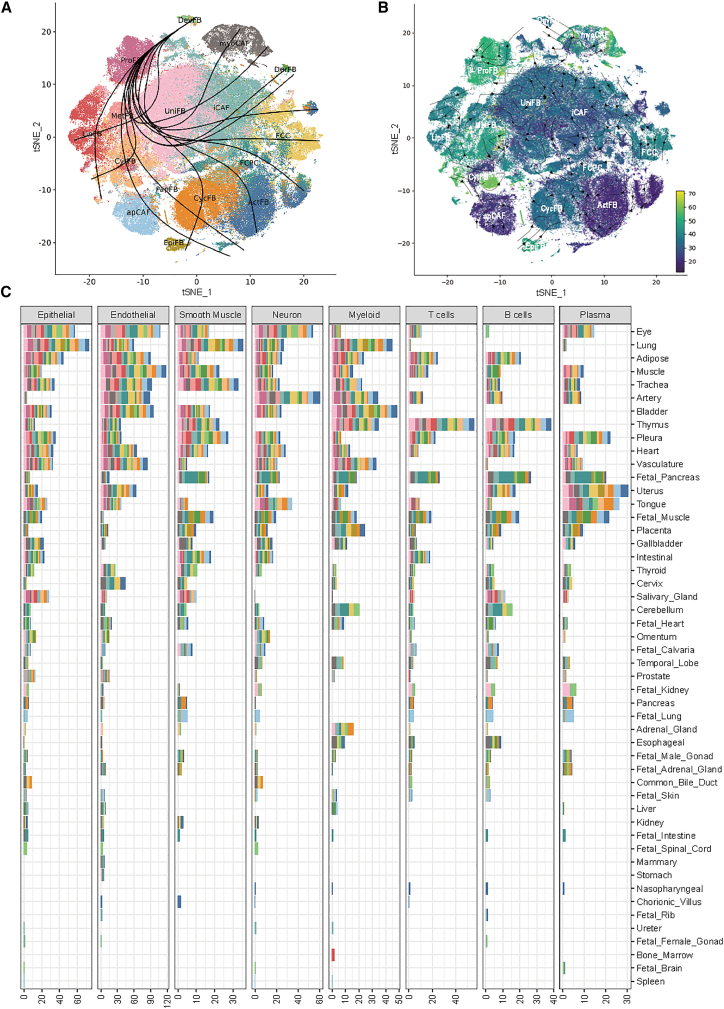
Characteristics of fibroblast subtypes (A) The t-SNE plot showing the trajectory analysis results by slingshot. Cells are colored by fibroblast subtypes. (B) The t-SNE plot showing the trajectory analysis results by PhyloVelo. Cells are colored by the pseudotime. The directions of the arrows show the trajectory of pseudotime. (C) Bar plot depicting the overall interactions of cell-cell communications. Bars are colored by fibroblast subtypes, with bar size representing interaction expression. *x* axis: the interactions between 16 fibroblast subtypes and 8 major cell types. *y* axis: tissues sorted by the overall interactions across 50 tissues.

To investigate the developmental dynamics and differentiation pathways of fibroblast subtypes, we conducted trajectory inference using three complementary methods to cross-validate our findings. First, we applied the Slingshot^36^ method to infer lineage trajectory and construct migration branches. As shown in Figure 2A, a total of 14 distinct trajectories were identified, revealing diverse differentiation directions among fibroblast cells. Notably, DevFB and ProFB were primarily located at the convergent root of all branches in the lower right region, suggesting that these subtypes represent early developmental stages. Second, we applied PhyloVelo^37^ to reconstruct cell trajectories and obtained a differentiation score to each cell. Consistent with Slingshot, DevFB and ProFB exhibited relatively high average velocity scores (DevFB = 34.126, ProFB = 48.446) (Figure 2B), indicative of a less differentiated, progenitor-like state. In contrast, apCAF and ActFB displayed lower average velocity scores (apCAF = 23.495, ActFB = 22.612) (Figure 2B), consistent with more differentiated stages. Finally, we applied Monocle^38^ to further characterize branching trajectories. Due to the large number of cells (*n* = 157,984), we randomly down-sampled to 15,798 cells, ensuring equal representation of fibroblast subtypes. This process was repeated 6 times to ensure robustness and reproducibility. Remarkably, ProFB consistently appeared at the root of inferred trajectory across all replicates (Figures S13A–S13F), reinforcing its potential role as an early progenitor population. We also studied the trajectory of fibroblast subtypes in the same tissues, respectively (Figure S13G). There were 46 tissues that had enough data for this analysis. This intra-organ comparisons highlighted both conserved and divergent gene expression programs and supported the notion that certain fibroblast states were developmentally regulated yet maintained organ-specific signatures. Taken together, all analyses indicated that DevFB and ProFB were at the early stages of development.

#### Cell-cell communication

We employed CellPhoneDB v.5^39^ to dissect the crosstalk between 16 fibroblast subtypes and the other 8 major cell types (Figures 2C, S14, and S15). The total interactions of ligand and receptor showed that there were intensive interactions among fibroblast subtypes, especially EpiFB, PapFB, ActFB, LipFB, MetFB, CytFB, and ProFB (Figure 2C). Most fibroblasts interacted with endothelial cells intensively, followed by epithelial cells (Figure S14B). However, fibroblast cells showed less interaction with myeloid or lymphocyte cells (T cells, B cells, plasma cells) (Figure S14A). Specifically, smooth muscle cells had the lowest interactions with fibroblast subtypes (Figure S14B). Moreover, we obtained and examined the top 5 ligand-receptor pairs for all cell types (Figure S15A). The pair *APP-CD74*, which could promote immunosuppression and progression of tumors,^40^ was the most frequently identified ligand-receptor pair among all interactions (12.8%, *n* = 82, Figure S15A). The communications among the fibroblast subtypes were also found to be mediated by specific interactions. For example, *COL1A1_integrin_a10b1_complex*, which belonged to adhesion by collagen/integrin, was unique to EpiFB (Figure S15A) and had been reported to regulate trophoblast invasion.^41^^,^^42^ Another pair, *APOE_TREM2_receptor*, which belonged to signaling by apolipoprotein*,* was unique to DerFB and was a risk factor for Alzheimer disease (Figure S15A).^43^ These specific interactions for each cell type further demonstrated the fibroblast heterogeneity.

Considering the heterogeneity of tissue niches, we further performed niche-relevant cell-cell communication (CCC) analyses across 56 tissues (Figure S15B). No detectable CCCs (defined by ligand-receptor pairs with gene expression >0.1) were observed in fetal stomach, fetal thymus, lymph node, rectum, skin, and fallopian tube (Table S11). Based on the overall number of interactions between the 16 fibroblast subtypes and 8 major cell types in the remaining 50 tissues, the tissues with the highest levels of CCCs included the eye, lung, adipose tissue, muscle, and trachea (Figure 2C). In these tissues, fibroblast subtypes showed more frequent interactions with non-immune cells (such as epithelial, endothelial, and smooth muscle cells) than with immune cells (including myeloid cells, T cells, B cells, and plasma cells) (Figure 2C). Notably, increased interactions were observed specifically among T and B cells in the thymus (Figure 2C). Furthermore, compared with adult/aged tissues, fetal tissues exhibited markedly fewer fibroblast-related interactions, indicating developmental differences in fibroblast communication dynamics (Figure 2C).

### Functional characteristics of fibroblast subtypes

The fine-mapped annotations of fibroblast subtypes allowed us to systematically explore the transcriptional heterogeneity of fibroblasts from aspects including cell states, functional enrichment, and regulatory networks.^44^

#### Cell states

**Figure 3:**
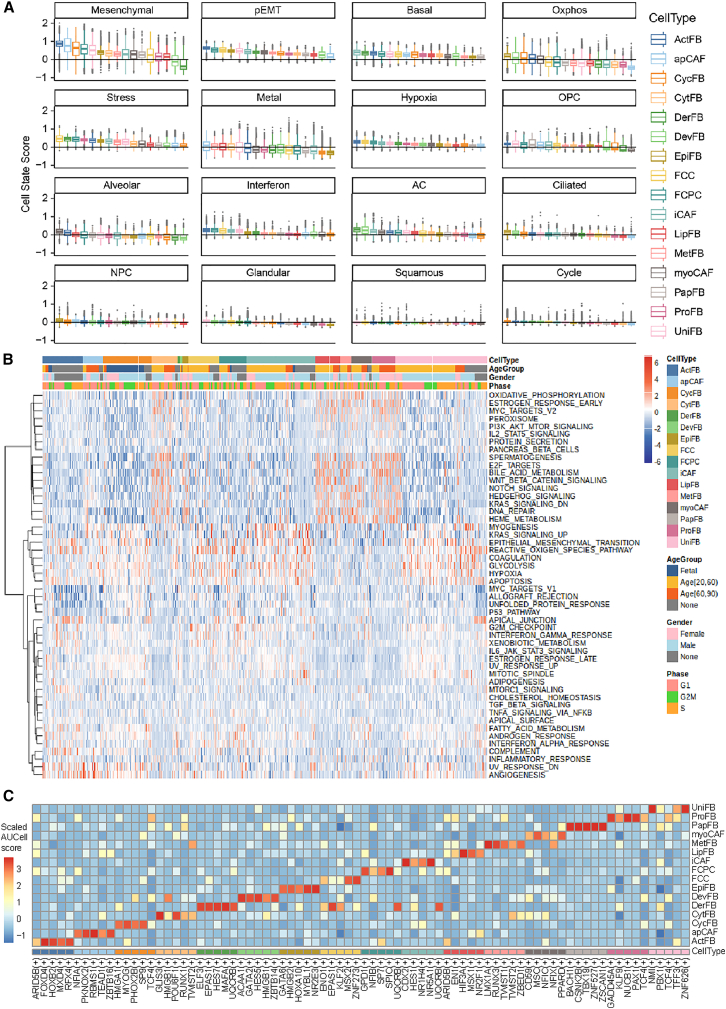
The mechanism and function of fibroblasts in distinct tissue (A) Boxplots showing the enrichment scores for each cell state colored by fibroblast subtypes. The boxes ware bounded by the first and third quartiles with a horizontal line at the median, and whiskers extend to the maximum and minimum value. (B) Heatmap of the VEGA results for the hallmark gene sets in each cell. The annotation color bar indicates the fibroblast subtypes. (C) Heatmap of the scaled AUCell score of the top 5 transcription factors in each fibroblast subtype. The annotation color bar indicates the fibroblast subtypes.

We collected 16 gene sets representing different cell states, such as stress, interferon response, and mesenchymal.^45^ We calculated scores for these cell states for each cell using the AddModuleScore function from Seurat. As shown in Figure 3A, four cell states showed substantial heterogeneity in different subtypes, which were the complete epithelial-mesenchymal transition (cEMT), metal reaction (metal), oxidative phosphorylation (oxphos), and stress response states (Figures 3A and S16A; Table S12). Within each fibroblast subtype, we observed both positive (co-occurring) and negative (mutually exclusive) correlations between cellular states. For example, hypoxia and partial EMT states consistently co-occurred across all 16 subtypes (mean SCC = 0.45, ranging from 0.23 to 0.64). In contrast, oxphos and stress states showed consistent negative correlations in all subtypes (mean SCC = −0.24, ranging from −0.45 to −0.08). Interestingly, the correlation patterns among cell states displayed subtype-specific directional differences (Figure S16B). For example, the cEMT state correlated positively with the stress state in FCC (SCC = 0.271, *p* value = 8.92 × 10^−119^) but negatively in DerFB (SCC = −0.456, *p* value = 7.46 × 10^−44^). Similarly, the oligodendrocyte progenitor cell (OPC) and stress states were positively correlated in PapFB (SCC = 0.307, *p* value = 1.91 × 10^−4^) but negatively correlated in DevFB (SCC = −0.322, *p* value = 3.05 × 10^−16^) (Figure S14B; Table S13). Furthermore, correlation heterogeneity was particularly evident when comparing subtypes. In some subtypes, strong and frequent correlations were observed among multiple states, e.g., UniFB showed 111 significant correlations (SCC *p* value <0.05) out of 16 × 15/2 = 120 possible pairs. In others, the correlations were much less, such as PapFB (61 significant correlations) and DerFB (66 significant correlations). This variability highlights the complex, context-dependent nature of cellular state interactions.

#### Functional enrichment by GSEA

We performed single sample gene set enrichment analysis (ssGSEA) analysis with hallmark gene sets from the Molecular Signatures Database (MSigDB) (access date: September 2022)^46^ to investigate pathway enrichment patterns for each cell. For better visualization, we implemented a semi-supervised hierarchical clustering method (ward.D2) by fixing cell type labels while grouping gene sets. As shown in Figure S17, two distinct groups of pathways were identified. The first group prominently included EMT, myogenesis, apoptosis, coagulation, and complement pathways, demonstrating consistent enrichment across most subtypes and validating fibroblasts’ essential roles in tissue remodeling, wound repair, and cytoskeletal maintenance. The second group was featured by the mTORC1 signaling pathway. Interestingly, ActFB and ProFB exhibited completely opposed activity patterns in both MYC targets V1 and oxidative phosphorylation pathways. These results further highlighted the transcriptional heterogeneity among the fibroblast subtypes (Figure S17).

#### Functional enrichment by VEGA

Considering the heavy sparsity of scRNA-seq data, we employed variational autoencoders enhanced by gene annotations (VEGA)^47^ to further investigate the functional characteristics of each subtype. VEGA is a deep learning method that incorporates gene-pathway annotations in one of its layers to enable interpretability and thus provides biological insights into the fibroblast subtypes. Importantly, VEGA can generate nonlinear embeddings of the input scRNA-seq data and provide alternative insights to the ssGSEA analyses. We ran VEGA on all 157,984 fibroblast cells using the same Hallmark gene sets from MSigDB, resulting in an embedding vector for each cell representing the activities for the 50 gene sets. Using the embedding matrix, we conducted the same semi-supervised hierarchical clustering as for ssGSEA results. As shown in Figure 3B, UniFB exhibited high activities of coagulation, EMT, glycolysis, hypoxia, and myogenesis, all of which were important factors for the construction of the ECM (Figure S18). Further examples included strong activities of the inflammatory response, UV response down, and angiogenesis in ActFB (Figure 3B) and E2F targets, heme metabolism, MYC targets v2, notch signaling, and WNT beta-catenin signaling in myoCAF, ProFB, LipFB, MetFB, and CytFB (Figures 3B and S18B). The results from both ssGSEA and VEGA revealed strong heterogeneities across fibroblast subtypes.

#### Regulatory networks

Transcription factors (TFs) play a pivotal role in regulating gene expression, contributing significantly to cellular heterogeneity.^48^ To systematically analyze TF activities and their target genes, we applied the single-cell regulatory network inference and clustering (SCENIC) method.^49^ SCENIC constructed regulons comprising TFs and their direct targets by integrating co-expression patterns with motif enrichment analysis, effectively eliminating indirect interactions. It calculated an AUCell score to assess the activity of each regulon. We applied SCENIC across all subtypes for 622 human TFs. Figure 3C showed the scaled AUCell scores for the top five most active TFs identified in each fibroblast subtype. Notably, the identified TFs exhibited functional coherence with their respective subtypes. For example, *MYOG*(+), known to regulate cell cycle and proliferation, was enriched in CycFB, a subtype characterized by active cell proliferation and primarily observed in fetal samples (Figure 3C). Similarly, *CDX2*(+), which drives a pro-inflammatory gene expression program, was specifically detected in iCAFs, aligning with their established roles in inflammation (Figure 3C). Strikingly, each subtype exhibited distinct major regulators, reflecting subtype-specific transcriptional programs. We identified six TFs shared across multiple subtypes, which were *ARID5B*(+) (ActFB and LipFB), *EPAS1*(+) (DerFB and FCC), *HMGB1*(+) (CytFB and DevFB), *TCF4*(+) (CycFB, ProFB, and UniFB), *TWIST2*(+) (CytFB and MetFB), and *UQCRB*(+) (DerFB, FCPC, and iCAF), implying that these TFs were involved in a relatively common regulatory program in fibroblasts (Figure 3C). We also expanded our analyses to eight non-fibroblast cell types using SCENIC.^48^ Specifically, we uncovered 80 cell type-specific TFs, including *EBF1*(+) in B cells, *NR2F1*(+) in endothelial cells, *TCF21*(+) in smooth muscle cells, INSM1(+) in epithelial cells, *BATF*(+) in T cells, *PRRX1*(+) in neurons, *XBP1*(+) in plasma cells, and *FOS*(+) in myeloid cells (Figure S19A). Furthermore, we identified 102 fibroblast-specific TFs that may serve as markers to distinguish fibroblasts from other major cell types (Figure S19B). Furthermore, 77 TFs were shared in fibroblast subtypes and non-fibroblast cell types. These results suggested both fibroblast-specific transcriptional signatures and conserved regulatory programs of cell types (Figure S19B).

### Cell pluripotency and cellular senescence of fibroblast subtypes

**Figure 4:**
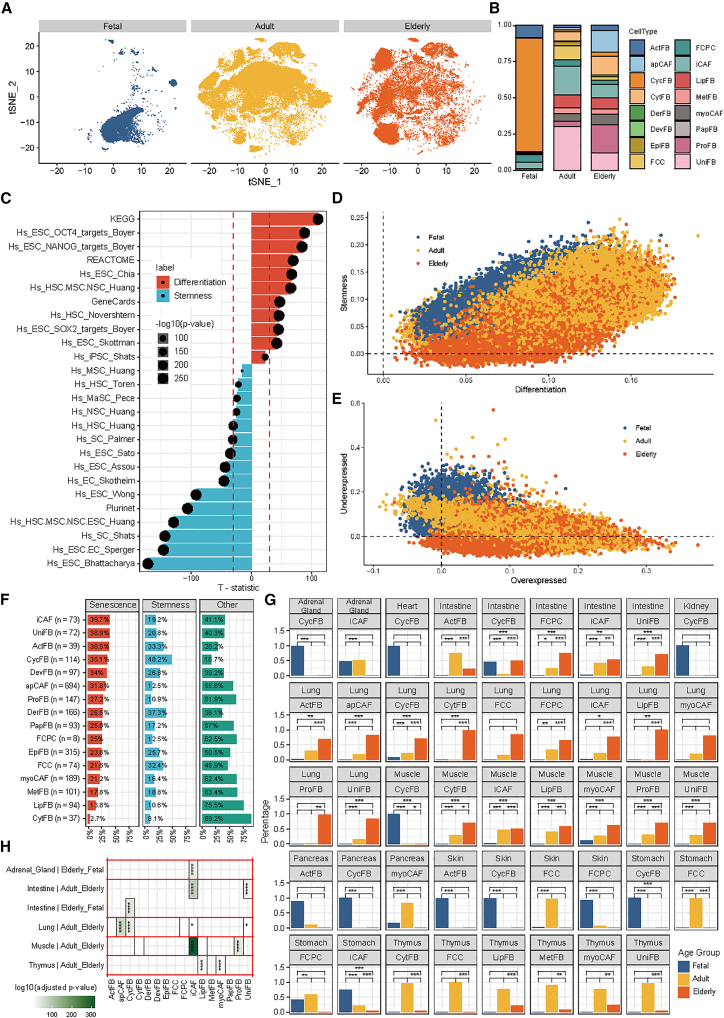
Cell pluripotency and cellular senescence of fibroblast subtypes (A) The t-SNE plot of fibroblast cells colored by age groups. Blue: fetal; orange: age from 20 to 60 years; yellow: age from 60 to 90 years. (B) The stacked bar plots of the percentage of fibroblast subpopulations in different age groups. (C) Bar plots showing the relationship between the 26 gene sets (Table S12) from the StemChecker database and age. *x* axis: the t-statistic from the linear regression model fitting. The dashed lines indicate where the t-statistic = 30 or −30. Dot size is proportional to −log10(*p* value). (D) The scatterplot of the stemness (*y* axis) and differentiation (*x* axis) of each cell, colored by age groups. (E) The scatterplot of the under-expressed genes (*y* axis) and over-expressed genes (*x* axis) of each cell, colored by age groups. (F) Overlap between fibroblast subtype DEGs and senescence gene sets. DEGs were identified using the FindAllMarkers function in Seurat for each subtype, using the criteria log2 (fold change) > 1 and adjusted *p* value <0.01. Red bar: percentage overlap with the senescence set (*n* = 4,353 genes). Blue bar: percentage overlap with the stemness set (*n* = 3,721 genes). Green bar: percentage of fibroblast subtype DEGs not overlapping with either set (other genes). (G) Bar plot showing whether the fibroblast subtypes were over-represented in age-related tissues (at least 100 fibroblast cells of each condition). The *p* value was calculated by Fisher’s exact test. ∗*p* value <0.05 and > 0.01; ∗∗*p* value <0.01 and > 0.001; ∗∗∗*p* value <0.001. (H) Comparisons of fibroblast subtypes among age groups (fetal and adult/elderly) in matched tissues (at least 100 fibroblast cells of each condition). The *p* values were calculated by a two-sided *t* test and corrected by the Benjamini-Hochberg method. ∗adjusted *p* value <0.05 and > 0.01; ∗∗adjusted *p* value <0.01 and > 0.001; ∗∗∗adjusted *p* value <0.001 and > 0.0001; ∗∗∗∗adjusted *p* value <0.0001.

To explore the developmental dynamics of fibroblast subtypes, we studied their age-dependent distribution patterns along with the pluripotency and senescence characteristics. We first stratified all samples into three age groups: fetal specimens (including unborn or aborted samples), adult specimens (20–60 years), and elderly specimens (≥60 years). As shown in Figure 4A, there were pronounced inter-group distinctions, particularly in fetal samples. Specifically, CycFB was dominant in fetal specimens but showed minimal presence in both adult and elderly groups (Figure 4B). Between the adult and elderly groups, FCC and UniFB decreased, whereas apCAF and ProFB increased with age (Figure 4B). Given these robust age-subtype associations, we subsequently examined cell pluripotency and senescence of fibroblast cells, which were important indicators of cellular aging.^50^^,^^51^

To assess pluripotency (encompassing stemness and differentiation), we collected 26 gene sets comprising 7,419 genes from the StemChecker database^52^ (Table S11). For each gene set, we calculated a module score and compared their difference among the three age groups by using the two-sided Wilcoxon test (Figure S20A) and fitting linear regression models. The beta coefficients from the regression models served as indicators of pluripotency status. As shown in Figure 4C, we observed that 10 gene sets (2,987 genes) exhibited age-related increases (beta >0, denoted as differentiation gene sets) and 11 gene sets (3,828 genes) showed age-related decreases (beta <0, designated as stemness gene sets) (|t-statistic| from the regression model >30). We collectively refer the 2,987 genes as the differentiation gene set and the 3,828 genes as the stemness gene set, each of which can be used to calculate a differentiation score and a stemness score for each cell (Figure 4D). As expected, the differentiation score of each cell increased with age (Figure S20B), whereas the stemness score decreased (Figure S20D). Particularly, fibroblast cells from the fetal specimens displayed elevated stemness scores (toward the top in Figure 4D). In addition, comparative analysis of subtypes revealed that tumor-like CAF populations (such as apCAF, iCAF, and myoCAF) exhibited particularly increasing stemness (Figure S20E). Interestingly, EpiFB displayed dual characteristics of elevated differentiation and stemness potential, consistent with their epithelial-mesenchyme plasticity (Figures S20C and S20E).

Senescent cells play important roles in human development and diseases.^53^ To systematically characterize senescence-associated transcriptional profiles, we retrieved the genes associated with cell senescence from the CellAge database,^54^ including 525 over-expressed and 734 under-expressed genes during cell senescence. We similarly calculated module scores for these two gene sets for each cell, where the over-expressed genes represented a high level of senescence and the under-expressed genes a low level of senescence, respectively (Figure 4E). Subtype-wise exploration of the senescence score showed that CycFB, which was dominantly found in fetal specimens, showed minimal expression of over-expressed marker genes and maximum expression of under-expressed marker genes (Figures S20G and S20I).

By exploring all four scores in 46,041 fibroblast cells, i.e., the differentiation scores (Figure S21A), the stemness scores (Figure S21B), the over-expressed scores (Figure S21C), and the under-expressed scores (Figure S21D), we found the differentiation and over-expressed scores showed similar performance (Figures 4D, S21A, and S21C), as did stemness and under-expressed scores (Figures 4E, S21B, and S21D). Thus, we combined differentiation and over-expressed gene sets to create a senescence signature (*n* = 4,353 genes, Figure S22A) and combined stemness and under-expressed gene sets to form a stemness signature (*n* = 3,721 genes) (Table S12). Comparison between the fibroblast subtype-specific markers and the senescence or stemness gene signatures showed that iCAF shared 73 genes (39.7% of iCAF) with the senescence signature genes and CycFB shared 114 genes (48.2% of CycFB) with the stemness signature genes (Figure 4F). This is consistent with the observation that CycFB was extremely prevalent in fetal tissues (Figure 4B).

By examining the fibroblast subtypes in three age groups in each tissue, we identified 10 tissues qualified for the following analyses (with at least one condition with >100 cells, Figures S22B–S22D). For each tissue, we tested if any fibroblast subtypes showed over-representation in different age groups (Figures 4F–4H) using Fisher’s exact test. Accordingly, CycFB was the most significantly enriched subtype in the fetal group in 7 tissues, including the adrenal gland, intestine, lung, muscle, pancreas, skin, and stomach. The iCAF showed an increasing percentage with age in intestine, lung, and muscle, i.e., fetal → adult → elderly (Figure 4G).

We next explored the senescence characteristics across different age groups for each subtype in each tissue. This analysis encompassed 16 conditions across 10 fibroblast subtypes distributed among 5 tissues (Figure S22E). As shown in Figure 4H, the iCAF subtype exhibited the age-related differences in 4 tissues: in the adrenal gland, iCAF showed higher senescence scores in the fetal group than in the elderly group (adjusted *p* value = 3.60 × 10^−26^); in the intestine, higher in adult than elderly (adjusted *p* value = 1.30 × 10^−83^); in the lung, higher in adult than elderly (adjusted *p* value = 4.80 × 10^−2^); and in muscle, higher in adult than elderly (adjusted *p* value = 1.90 × 10^−318^). This indicates that iCAF subtypes show greater senescence heterogeneity across the lifespan (Figure 4H). This also aligns with the high proportion of senescence signatures observed in iCAF (Figure 4F). In the lung, apCAF showed significantly higher senescence scores in the elderly than in the adult group (adjusted *p* value = 6.50 × 10^−67^) (Figure 4H).

### The well-defined fibroblast subtypes unveiled cell-type compositions critical for clinical outcomes

**Figure 5:**
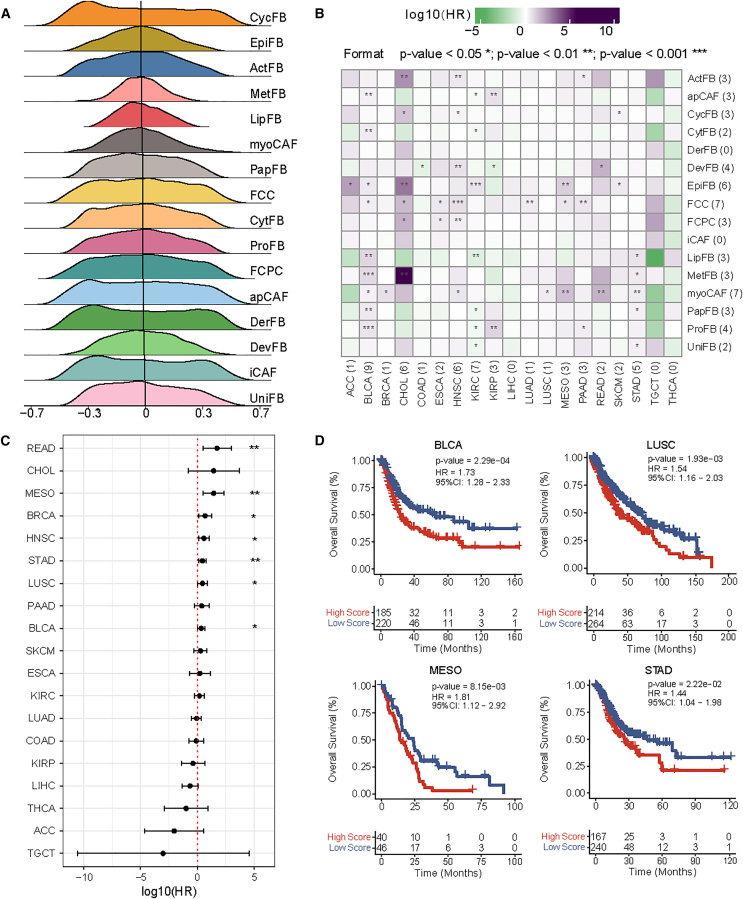
The well-defined fibroblast subtypes unveiled cell-type compositions critical for clinical outcomes (A) The density plots of 16 fibroblast subtypes of TCGA samples. *x* axis: the enrichment score. The color represents the fibroblast subtypes. (B) The survival status of 16 fibroblast subtypes in TCGA cancer types. The *p* value and HR were calculated by multivariate Cox proportional hazards regression models using age, sex, tumor stage, pathology N stage, and pathology T stage as covariates. The color is proportional to the log10(HR) (green indicating log10(HR) < 0 and purple indicating log10(HR) > 0). ∗*p* value <0.05 and >0.01; ∗∗*p* value <0.01 and >0.001; ∗∗∗*p* value <0.001. (C) The forest plot showing log10(HR) (*x* axis) in 33 TCGA cancer types obtained from the multivariate Cox models using the myoCAF composition to stratify samples. The lines are bounded by the lower and higher 95% confidence interval (CI). ∗*p* value <0.05 and >0.01; ∗∗*p* value <0.01 and >0.001; ∗∗∗*p* value <0.001. (D) The survival plots of samples stratified by the myoCAF compositions in BLCA, LUSC, MSEO, and STAD. The samples were divided into high- and low-score groups by the mean enrichment score (red: high-score group; blue: low-score group). The *p* value, HR, and 95% CI were calculated by log rank survival analysis. The full names of cancer types are available in Table S12.

The well-defined fibroblast subtypes also allowed us to infer cell type compositions in bulk RNA-seq data and explore their associations with clinical outcomes. Using subtype-specific DEGs as gene sets, we calculated enrichment scores using GSVA^55^ for each set in each sample across 33 cancer types from TCGA. The density of the enrichment scores for 16 fibroblast subtypes is present in Figure 5A. For each cancer type (full name available in Table S8), we categorized the samples into two groups by the mean value of the enrichment scores across samples and conducted the survival analyses (supplemental information). We identified 72 fibroblast subtype and cancer type associations (log rank test, *p* value <0.05) (Figure S23; Table S14), among which 53 remained significant using the multivariate Cox proportional hazards regression model adjusting for clinical covariates (age, sex, tumor stage, pathology N stage, and pathology T stage) (*p* value <0.05, Figure 5B). Among these associations, the subtype myoCAF most frequently distinguished outcomes (*p* value <0.05 in BLCA, BRCA, HNSC, LUSC, MESO, READ, and STAD, Figure 5B), while BLCA showed the broadest subtype associations (9 fibroblast subtypes, Figure 5B). In BRCA, iCAF and myoCAF showed opposing prognostic effects, where a lower iCAF composition was correlated with a favorable outcome (log rank test: hazard ratio [HR] = 0.672, *p* value = 0.016; Cox test: HR = 0.659, *p* value = 0.175; Table S14), whereas a higher myoCAF composition was associated with worse prognosis (log rank test: HR = 1.279, *p* value = 0.130; Cox test: HR = 4.949, *p* value = 0.013; Figure 5B). Overall, myoCAF was significantly associated with seven cancer types as a risk factor (READ, MESO, BRCA, HNSC, STAD, LUSC, and BLCA, Figure 5C, ordered by decreasing HR), with four (MESO, STAD, LUSC, and BLCA) supported by the log rank test (*p* value <0.05, Figure 5D). Additional survival analyses for other fibroblast subtypes, including iCAF and apCAF, were shown in Figures S23 and S24. These results were consistent with previous reports linking increased myoCAF composition to adverse outcomes.^22^^,^^56^

### The well-defined fibroblast subtypes unveiled cell-type contexts for complex traits

The well-annotated fibroblast subtypes can also help us understand the cellular mechanisms underlying complex diseases.^57^ We collected genome-wide association studies (GWASs) summary statistics for 74 traits (Table S15) and applied two complementary methods to identify the fibroblast subtypes that exhibited excessive expression of trait-associated genes at both cell-type and single-cell resolutions: the decoding cell-type specificity (deCS) method^58^ and the single-cell disease relevance score (scDRS) method.^59^ For both methods, we first defined trait- or disease-associated genes by using multi-marker analysis of genomic annotation (MAGMA)^60^ method. MAGMA maps SNPs from GWAS summary statistics to genes using an annotation window of 10 kb and calculates a *Z* score for each gene.

**Figure 6:**
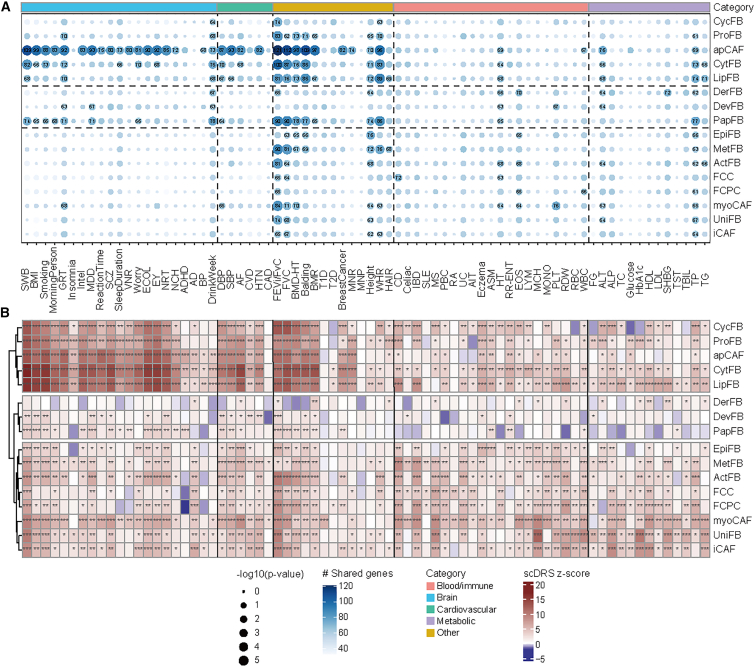
The well-defined fibroblast subtypes unveiled cell-type contexts for complex traits The association results for fibroblast subtypes and 74 complex traits or diseases assessed by deCS (A) and scDRS (B). Dot size is proportional to Fisher’s exact test −log10(*p* value) from deCS. Dot color is proportional to the shared genes between subtype-specific DEGs and disease-associated genes from deCS. The full names of traits are available in Table S14.

deCS employs trait-associated genes (top 1,000 genes per trait) to assess their significant over-representation in cell-type-specific genes through Fisher’s exact test. As a result, deCS identified 169 significant associations (unadjusted *p* value <0.05) out of 74 traits × 16 subtypes (total 1,184 tests). Similarly, scDRS uses the top 1,000 trait-associated genes as defined by MAGMA and conducts cell-level enrichment tests, detecting 361 significant associations (unadjusted *p* value <0.05). Notably, 106 associations were concordant between both methods, demonstrating significant overlap beyond random expectation (Fisher’s exact test, *p* value = 1.201 × 10^−13^, Table S16), confirming the robustness of these trait-subtype associations. Both methods consistently highlighted that apCAF, CytFB, LipFB, and PapFB were the most frequently enriched cell types, particularly in brain and heart tissues (Figures 6A and 6B; Table S16). Several cardiac-related traits, e.g., diastolic blood pressure (DBP)/systolic blood pressure (SBP), atrial fibrillation (AF), cardiovascular diseases (CVD), and hyperthension (HTN) (full name available in Table S15), showed enrichment across multiple fibroblast subtypes, including CycFB, ProFB, apCAF, CytFB, and LipFB by the scDRS method, with most findings corroborated by deCS (Table S16). Additionally, lung diseases (forced expiratory volume in 1 Second / forced vital capacity (FEVi/FvC) and forced vital capacity (FVC)) were enriched with multiple subtypes (Figures 6A and 6B). These findings collectively advanced our understanding of the molecular mechanisms of complex diseases at the cellular resolution.^61^

## Discussion

In this study, we conducted a systematic analysis of scRNA-seq data from diverse human tissues and organs across multiple biological conditions, encompassing normal, disease, abortion, and cancer. Our work established an integrative cellular atlas of fibroblasts, identifying 16 distinct subtypes, including 10 previously characterized and six putatively not reported subtypes. By thorough characterization, we revealed the unique expression patterns and heterogeneity of these fibroblast subtypes from various aspects, including cell states, gene regulation network, cell pluripotency, and cellular senescence. With the systematic annotation and definition of marker genes, we further demonstrated the clinical relevance of these fibroblast subtypes by inferring their relationships with prognosis and unveiling the cellular contexts of complex tissues. Overall, we present a systematic investigation of a single-cell transcriptional atlas of fibroblasts and their subtypes in human tissues. The well-annotated fibroblast subtypes serve as a valuable resource for future research.

Characterizing a specific cell type, such as fibroblasts, presents significant challenges, particularly given the rapidly growing and continuously evolving collection of publicly available scRNA-seq datasets. In our study, to reduce the noise, we adopted a stringent strategy to define fibroblast cells by requiring supporting evidence from both NES^62^ and CellTypist^57^ upon the batch-corrected data and further validating using the well-known fibroblast marker genes, *COL1A1* or *COL1A2*. Thus, we considered the data and information on fibroblasts were of high quality in this study. We examined fibroblast subtypes over multiple developmental stages, tissues, cancer types, and diverse disease or normal conditions to ensure that we covered all possible types of fibroblast subtypes. Importantly, among the 16 subtypes, seven subtypes were considered not reported as they have not been reported in literature yet: CycFB, CytFB, DevFB, EpiFB, MetFB, and ProFB. CycFB was mainly identified in abortion (i.e., fetal) tissues. Its active regulon was regulated by the transcriptional factor *MYOG*, which was involved in cell cycle and proliferation, supporting that CycFB was indeed active in early developmental stages. DevFB and ProFB were found at the early branches of fibroblast evolutionary trajectories. Note that DevFB and ProFB were identified in all types of tissues, indicating that their location in the trajectory branches was not inflated by the developmental stages. EpiFB, as the name suggested, was involved in collagen adhesion, EMT processes, and the regulation of trophoblast invasion.^41^^,^^42^ The remaining three subtypes, ActFB, CytFB, and MetFB, were enriched in the E2F targets, heme metabolism, MYC targets v2, notch signaling, and WNT beta-catenin signaling pathways, implying a high metabolic activity.

These well-defined fibroblast subtypes provided opportunities to decode disease-relevant cell types and regulatory mechanisms. Integrating these fibroblast subtypes with GWAS summary statistics data, we found that multiple complex traits and diseases were associated with various subtypes, including Drinks per Week (genes highly expressed in 8 subtypes by deCS and 5 by scDRS), FEV1-forced vital capacity (FVC) ratio (14 by deCS and 8 by scDRS), FVC (12 by deCS and 7 by scDRS), height (12 by deCS and 3 by scDRS), and waist-hip ratio (WHR) (9 by deCS and 6 by scDRS). In cancer, CAF has long been recognized as an essential component of TME and is involved in multiple processes, including interaction with the immune cells and affecting the outcome of immunotherapy.^58^ Two subtypes of CAF, i.e., iCAF and myoCAF, both of which have been replicated in our study, were found to be particularly associated with different clinical outcomes. For example, samples with increased myoCAF percentage tended to have adverse survival.^22^^,^^56^ From the aspect of subtypes, CycFB, ProFB, apCAF, CytFB, and LipFB were significantly associated with many diseases. Interestingly, CycFB has high stemness properties and could be a candidate cell type for reversing aging.

### Limitations of the study

There are several limitations of our study. First, we did not use data from other omics to verify the regulatory patterns of fibroblast subtypes. With the development of single-cell technologies, other types of omics data can be included in the future to verify the current results and further illustrate biological regulation and other mechanisms. Second, it is necessary to verify the fibroblast subtypes *in vivo* or *in vitro*.^59^ Third, cross-species studies will further support fibroblast characteristics and functions. Large-scale, pan-organ, and pan-tissue data are expected to be available soon. Fourth, more investigation is required to understand the functions of subtypes in cancer tissues, especially their relationships with clinical outcomes.

## Resource availability

### Lead contact

Further information and requests for resources and reagents should be directed to and will be fulfilled by the lead contact, Peilin Jia (pjia@big.ac.cn).

### Materials availability

This study did not generate new reagents.

### Data and code availability

• Data: The gene expression datasets generated during this study were publicly available and can be downloaded through GEO web portal using accession listing GSE109816,^60^ GSE121893,^60^ GSE123813,^61^ GSE130148,^63^ GSE131907,^64^ GSE132257,^65^ GSE144735,^65^ GSE145137,^66^ GSE150430,^67^ GSE153643,^68^ GSE158127,^69^ GSE159929,^70^ GSE160269,^71^ GSE171524,^72^ GSE134355^73^ (Human Cell Landscape, HCL), and GSE201333^74^ (Tabula Sapiens).
• Code: The bioinformatic analysis codes and source data were uploaded to GitHub (https://github.com/zxl2014swjx/Fibroblast) and Figshare (https://figshare.com/articles/dataset/Fibroblast_Heterogeneity/29582057) and are publicly available.
• Other items: All data needed to evaluate the conclusions of the current study were present in the manuscript and the supplemental information.

## Acknowledgments

This research was supported by the 10.13039/501100001809National Natural Science Foundation of China (NSFC) (92374103 and 32270706) and the Strategic Priority Research Program of the 10.13039/501100002367Chinese Academy of Sciences (XDB38010400). We would like to thank the authors for generating and publicly sharing the sc(n)RNA-seq data used in this study, which made our study feasible.

## Author contributions

X.Z., Z.Z., and P.J. conceived the study. X.Z. acquired, analyzed, and interpreted the data. X.Z., H.K., Z.Z., and P.J. wrote the manuscript. P.J. provided funding support. X.Z., H.K., Z.Z., and P.J. reviewed and approved the final version.

## Declaration of interests

The authors declare no competing interests.

## STAR★Methods

### Key resources table

**Table:** 

REAGENT or RESOURCE	SOURCE	IDENTIFIER
**Software and algorithms**		

R	CRAN	https://cran.r-project.org/src/base/R-4/R-4.1.1.tar.gz
Clustree	Package (version 0.4.4)	https://cran.r-project.org/web/packages/
ROGUE	Package (version 1.0)	https://cran.r-project.org/web/packages/
Slingshot	Package (version 2.2.0)	https://cran.r-project.org/web/packages/
Monocle	Package (version 2.22.0)	https://cran.r-project.org/web/packages/
CIBERSORT	Package (version 0.1.0)	https://cran.r-project.org/web/packages/
clusterProfiler.	Package (version 4.2.2)	https://cran.r-project.org/web/packages/
GSVA	Package (version 1.42.0)	https://cran.r-project.org/web/packages/
Survival	Package (version 3.2.11)	https://cran.r-project.org/web/packages/
Survminer	Package (version 0.4.9)	https://cran.r-project.org/web/packages/
Ggpubr	Package (version 0.4.0)	https://cran.r-project.org/web/packages/
ggplot2	Package (version 3.5.2)	https://cran.r-project.org/web/packages/
Stats	Package (version 4.1.1)	https://cran.r-project.org/web/packages/
deCS	Package (version 1.0)	https://cran.r-project.org/web/packages/
Python	Python (version 3.7.12)	https://www.python.org/downloads/release/python-3712/
PhyloVelo	Version 0.1	https://github.com/kunwang34/PhyloVelo
scDRS	Version 1.0.3b	https://github.com/martinjzhang/scDRS

**Deposited data**		

Code Repository	This paper	https://github.com/zxl2014swjx/Fibroblast
Data Repository	This paper	https://figshare.com/articles/dataset/Fibroblast_Heterogeneity/29582057

### Experimental models

This study did not involve experimental models.

### Method details

#### Single-cell RNA sequencing data

Datasets GSE109816^60^ and GSE121893.^60^ The original study conducted single-cell RNA sequencing (scRNA-seq) for samples with cardiomyocytes and non-cardiomyocytes from normal, failed, and partially recovered adult human hearts. Transcriptome profiling of 21,422 single cells was provided, with annotation information for age, gender, and cell types.

Dataset GSE123813.^61^ The original study collected site-matched tumors from basal and squamous cell carcinoma before and after anti-PD-1 therapy (Pembrolizumab and Cemiplimab) with ICI response and conducted scRNA-seq (*n* = 79,046 cells). It included pre- and after- treatment patients and left- and right- basal sites.

Dataset GSE130148.^63^ The original research aimed to chart the landscape of the upper and lower airways. The dataset contained scRNA-seq data (*n* = 10,360 cells) for healthy and asthmatic lungs (provide sample sizes).

Dataset GSE131907.^64^ The original study conducted scRNA-seq for normal and cancer (early and advanced tumor stage, lymph node and brain metastases) lungs from 44 patients (*n* = 208,506 cells). The 9 major cell lineages of lungs included stromal, immune, and gliocyte.

Dataset GSE132257^65^ and GSE144735.^65^ The two studies conducted scRNA-seq using the colorectal tissues from the normal mucosa and primary colorectal cancer patients (CRC) with the accession ID GSE132257 (*n* = 18,409 cells) and GSE144735 (*n* = 27,414 cells). The annotated CRC landscape contained 6 major cell types. The clinical information was available, including microsatellite instability (MSI) and consensus molecular subtypes (CMS) annotations.

Dataset GSE145137.^66^ The original study conducted scRNA-seq on a single case of chemotherapy-resistant, muscle-invasive urothelial bladder cancer (MIUBC) (*n* = 2,075 cells).

Dataset GSE150430.^67^ The original study conducted scRNA-seq to reveal the landscape of nasopharyngeal carcinoma (NPC) (*n* = 48,584 cells). A total of 15 NPC tumors and 1 normal sample were included in this dataset. The immune-related signatures were predicted to improve clinical outcomes in NPC.

Dataset GSE153643.^68^ Muus et al. conducted scRNA-seq using the adipose and liver tissues from severe acute respiratory syndrome coronavirus 2 (SARS-CoV-2) infection. It was a part of the GSE153643 datasets (*n* = 24,250 cells) of adipose and liver tissues.

Dataset GSE158127.^69^ Lung tissues from three lung transplantation patients with severe nonsolving COVID-19-associated respiratory failure were investigated using scRNA-seq. The dataset included 269,809 cells for lung tissues.

Dataset GSE159929.^70^ This dataset included scRNA-seq data (*n* = 84,363 cells) for 15 major tissues, all of which were from a male donor who died of traumatic brain injury. The tissues included the bladder, blood, common bile duct, esophagus, heart, liver, lymph node, bone marrow, muscle, rectum, skin, small intestine, spleen, stomach, and trachea.

Dataset GSE160269.^71^ The original study conducted scRNA-seq for Zhang X. et al. (2021) provided 60 esophageal squamous-cell carcinoma (ESCC) cases with TNM stage and 4 adjacent normal esophageal individuals (*n* = 208,659 cells). Additionally, the smoke and drink status were downloaded from the Supplemental data. The original study identified 8 major cell populations, including CD45^−^cells (epithelial cells, fibroblasts, endothelial cells, pericytes, and fibroblastic reticular cells) and CD45^+^ cells (T cells, B cells, and myeloid cells).

Dataset GSE171524.^72^ The original study conducted single-nucleus RNA sequencing (snRNA-seq) for the lethal lung tissues obtained from 21 COVID-19 patients who died of the disease. A total of 116,314 cells were obtained and used to identify cellular composition, cell states, and cell-to-cell interactions to improve understanding of long-term complications of severe SARS-CoV-2 infection.

Dataset GSE134355.^73^ The original study generated a comprehensive human cell landscape (HCL) containing more than 60 tissues and 599,926 cells from fetal and adult donors.

Dataset GSE201333.^74^ This dataset is also called the Tabula Sapiens dataset for humans. It included scRNA-seq data for 24 tissues from 15 donors (*n* = 483,152 cells) with age and gender information. The scRNA-seq data were divided into four main cell types, i.e., immune cells, epithelial cells, endothelial cells, and stromal cells.

#### Single-cell and single-nucleus RNA-seq data preprocessing

To analyze the distribution of fibroblast cells in different tissues and biological conditions, we collected a comprehensive dataset including 2,195,794 cells from 16 scRNA-seq or snRNA-seq studies representing 60 major human organs or tissues (Tables 1 and S1). These data were generated from 477 samples representing different biological conditions, including age, sex, smoking, and drinking, among others. The original samples can be divided into four categories: normal, disease, cancer, and abortion (Tables 1 and S1).

The workflow of data processing is shown in Figure S1. We required each sample to have at least 2000 cells (Tables 1 and S1), resulting in 368 samples from 14 cohorts encompassing 2,062,895 cells. Furthermore, we used Scanpy to exclude genes expressed in less than 3 cells and to filter out cells with less than 200 genes or with a high fraction of counts from mitochondrial genes, resulting in 2,034,315 cells (Figures S2A–S2D). To remove potential batch effects, we applied the harmony analysis using the top 2000 highly variable expressed genes (HVGs) (Figures S2G and S2H) and obtained batch-corrected data (Figure S2E, before harmony; Figure S2F, after harmony). Afterward, we applied the Louvain algorithm and classified all cells into 20 clusters (Figure S3A). The similarity among clusters was assessed by using the Spearman correlation coefficient (SCC) (Figure S3B) and dendrogram (Figure S3C). Subsequently, t-distributed Stochastic Neighbor Embedding (t-SNE) was employed to reduce dimension and visualize the cell clusters in a two-dimensional space (Figure S3A). By reviewing the dendrogram and the expression levels of well-known representative marker genes (e.g., *CD79A* and *MS4A1* for B cells, *GNLY*, *NKG7*, and *IL7R* for T cells, etc.) (Figure S3D) as well as the cluster-specific genes (Figure S3E), we merged the 20 clusters into 9 major cell types. They were myeloid cells (17.8% of total cells, *n* = 361,099 cells), B cells (7.0% of total cells, *n* = 141,400 cells), T cells (CD4^+^ T cells, CD8^+^ T cells, and NK cells) (24.5% of total cells, *n* = 497,892 cells), plasma cells (2.8% of total cells, *n* = 57,366 cells), epithelial cells (20.8% of total cells, *n* = 423,326 cells), endothelial cells (6.1% of total cells, *n* = 123,411 cells), neuron cells (2.9% of total cells, *n* = 59,833 cells), smooth muscle cells (1.1% of total cells, *n* = 22,300 cells), and fibroblast cells (17.1% of total cells, *n* = 347,688 cells) (Figures 1A and S9C; Table S2).

As a result, we obtained 2,062,895 cells from 368 samples from 14 studies. These cells were categorized into 9 major cell types: myeloid cells, B cells, T cells, plasma cells, epithelial cells, endothelial cells, neurons, smooth muscle cells, and fibroblast cells (Figures 1A, S1, and S9C; Table S2). We next focused on the fibroblasts (Figures S4–S8; Tables S3, S4, S5, and S6). Among the 9 cell types from our analyses, there are 347,688 fibroblast cells. In contrast, using the metadata from each of the original studies, we identified 256,711 pre-defined fibroblasts (Table 1). Considering the inconsistency between the two lines of annotation, i.e., our “harmony pipeline identified” fibroblasts and the pre-defined fibroblasts (overlapping *n* = 223,476 cells), we further implemented two ways of filtering strategies. Firstly, we curated a fibroblast gene set consisting of 538 fibroblast-related markers^26^ and conducted Gene Set Enrichment Analysis (GSEA).^62^ Normalized enrichment score (NES) was calculated for each cell using the fibroblast signature set. Secondly, we applied CellTypist^57^ to annotate the cell types for each cell. Applying to our harmony-identified fibroblasts, we retained cells that had NES >1.0 (*n* = 302,202 cells) and were annotated as fibroblasts by CellTypist (*n* = 176,940 cells), resulting in 157,984 fibroblast cells for further analysis (45.4% of 347,688 *harmony*-identified fibroblasts, 55.9% of 256,711 pre-defined fibroblasts (Table 1; Figure S1).

We next took two strategies to verify the resultant fibroblast cells. First, we explored multiple features of these fibroblasts in the original datasets, including the number of genes, the total number of counts for a cell, the proportion of mitochondrial read counts, and the proportion of total counts for a cell that were ribosome-associated (Figures S4A–S4D), confirming that there was no outstanding feature to drive the cell classification. Second, we explored the expression of marker genes for fibroblasts. Among the top 20 HVGs, 60% were previously reported marker genes for fibroblast (Figure S4E; Table S3).^26^ We also verified that the majority of fibroblast cells expressed the two well-known fibroblast markers, *COL1A1* and *COL1A2* (83.4% of cells had a count value >1 for *COL1A1* or *COL1A2*) (Figure S4F).

With the evaluation of the fibroblast cells, we next defined the subtypes of fibroblast cells. We applied clustree^24^ with the Louvain algorithm, followed by assessment using the Ratio of Global Unshifted Entropy (ROGUE)^25^ algorithm. Branches of clusters generated at different resolution values from 0 to 3 were shown in Figure S5A, and the t-SNE plots were displayed in Figure S5B for the clusters at each resolution value. The local inverse Simpson’s index (LISI)^75^ showed that at the resolution = 2.3, the clusters exhibited the highest LISI than the clusters obtained at other resolution values, indicating better separation of the clusters and well-controlled batch effect at this resolution value (Figure S5C).^76^ We also applied ROGUE to fine-map the appropriate resolution values and found that at resolution = 2.3, the median value of ROGUE reached the highest, resulting in 41 clusters (Figure S5D).

For each of the 41 clusters, which can be visualized in Figures S6A and S6B, we explored their distribution in each tissue of origin and found that many cell types were enriched in specific tissues (Figure S6C), e.g., clusters 16 and 19 enriched in adipose and clusters 4 and 5 enriched in fetal-related tissues. We then identified the marker genes (Figure S7A) for each cluster and merged those that shared at least 5 of the top 20 DEG (Table S3).^76^ For example, clusters 1 and 28 shared 5 DEG genes, although the two clusters were enriched in different tissues (Figure S7B) and were merged. After this step, the 41 clusters were merged into 25 clusters. Clusters were further merged if the average ROGUE was higher than each cluster individually. Names of these subtypes were based on recommendations from CellMarker2 or their tissue origin (Figure S8–S10; Tables S4 and S5).^26^

#### Classification of fibroblast subtypes

We used clustree^24^ with the Louvain algorithm to identify the subclusters of the fibroblast group. As for the batch effect, we used the local inverse Simpson’s index (LISI) to estimate the results of removing the batch effect. The LISI defines the effective number of datasets near a single cell of a class.^75^ The higher the LISI value, the more datasets in the neighborhood, and the smaller the batch effect. To assess the purity quantification of each single cluster, we employed ROGUE,^25^ which has been proven robust and independent of methods used for normalization, dimensionality reduction, and clustering. Thus, we used ROGUE to guide the splitting (re-clustering) and merging of the subtypes of fibroblasts to finally determine the fibroblast subtypes.

#### Evaluation of the differentiation potential of different single-cell subsets

A pseudo-time analysis was implemented to construct the evolutionary trajectories and understand the developmental trend of fibroblast subtypes. Specifically, we applied three complementary methods, i.e., Slingshot,^36^ PhyloVelo,^37^ and Monocle,^38^ to investigate the development and differentiation of fibroblast subtypes. For all the trajectory analyses, we utilized the normalized expression data from each sample.

#### Deconvolution of TCGA bulk RNA-seq by CIBERSORT

The reference signature matrix was constructed based on the average gene expression profiles of 8 major cell types and 16 fibroblast subtypes. mRNA expression data and corresponding clinical information from TCGA were obtained via cBioPortal.^77^ Deconvolution was performed using CIBERSORT^35^ with default parameters, except that the number of permutations was set to 100, as recommended by the authors to enhance robustness.

#### Functional enrichment by GSEA and VEGA

To explore the functional gene sets that are enriched in each fibroblast subtype, we applied GSEA from the R package clusterProfiler. The Hallmark gene sets were downloaded from MSigDB. Multiple test correction was implemented by the Benjamini & Hochberg (BH) method.

Variational autoencoders enhanced by gene annotations (VEGA) reduced dimensions for a sparse architecture and increased interpretability by using gene module activities.^47^ The input data of VEGAS included a count matrix and an annotation file for the Hallmark gene sets, requiring set sizes between 20 and 1,000 genes. The dropout rate was 0.5. The learning rate was 1 × 10^−4^. The epoch was set as 15, as the loss value started to increase after this value (Figure S18A). The training patience was set as the default value of 10.

#### Single-cell regulatory network inference and clustering analysis

We employed SCENIC to dissect the active TFs of each fibroblast subtype. A motif sequence validation statistical method was introduced to infer gene co-expression networks to identify highly reliable transcription factor networks. For each fibroblast subtype, we used three steps to assess the activity of related regulons (TF and target genes) in individual cells, respectively. First, the GRNBoost function infers co-expression modules including a potential TF and its target genes. Second, the RcisTarget function identifies the motifs with correct upstream regulators significantly enriched in each regulon. Third, the AUCell score is calculated for each regulon to present the activities.

#### Analyses of TCGA bulk RNA-seq datasets

The mRNA expression counts and clinical information from TCGA were downloaded from cBioPortal.^77^ GSVA^55^ was used to calculate an enrichment score for each fibroblast subtype in each sample using the bulk expression data (log10-transformed). The gene sets of 16 fibroblast subtypes were generated by using the subtype-specific differential expression genes (DEGs).

Survival analyses and visualization were conducted using the R packages Survival and Survminer. The log rank test was implemented by the survdiff function from the R package Survival. The multivariate Cox proportional hazards regression method was implemented by using the coxph function from the R package Survival. The Kaplan-Meier (KM) plots were created by using functions from the R packages Survival and Survminer.

### Quantification and statistical analysis

All software used has been listed in the key resources table. The descriptions of statistical tests were specified in the corresponding figure legends. All statistical analyses were performed using R (version 4.1.1).

## Supplemental information

Document S1. Figures S1–S24

Table S1. Clinical characteristics of collected samples

Table S2. Statistics about the 9 major cell types

Table S3. Statistics of the differentially expressed genes for each cell cluster

Table S4. The annotations of fibroblast subtypes

Table S5. The marker genes for fibroblast subtypes

Table S6. Statistics of fibroblast subtypes in collected cohorts

Table S7. The previous fibroblast categorizations

Table S8. The bulk RNA-seq expression datasets from the TCGA database

Table S9. The full name of the cancer types, related to Figures 1F and S12D

Table S10. The significant enrichment of fibroblast subtypes in disease or cancer

Table S11. The cell-cell communications between 16 fibroblast subtypes and 8 major cell types in the tissue niche

Table S12. The marker genes of cell stemness, cellular senescence, cell state, and DNA damage repair pathways

Table S13. The correlation among 16 cell states in 16 fibroblast cell types

Table S14. Results of survival analysis for TCGA bulk RNA-seq data

Table S15. Information about the GWAS traits obtained from previous works and the UKBB database

Table S16. Results of fibroblast subtype enrichment analyses for complex traits by using deCS and scDRS
