# Supplementary material for: A systematic characterization of fibroblast subtypes and heterogeneity
Source: iScience. 2025 Nov 10;28(12):113915. doi: 10.1016/j.isci.2025.113915 (PMC12686731; doi:10.1016/j.isci.2025.113915)
Supplement: Document S1. Figures S1–S24 [file mmc1.pdf]

iScience, Volume *28*

Supplemental information

A systematic characterization of fibroblast subtypes and heterogeneity

Xiuli Zhu, Hongen Kang, Zhongming Zhao, and Peilin Jia

Supplemental Figures and Legends

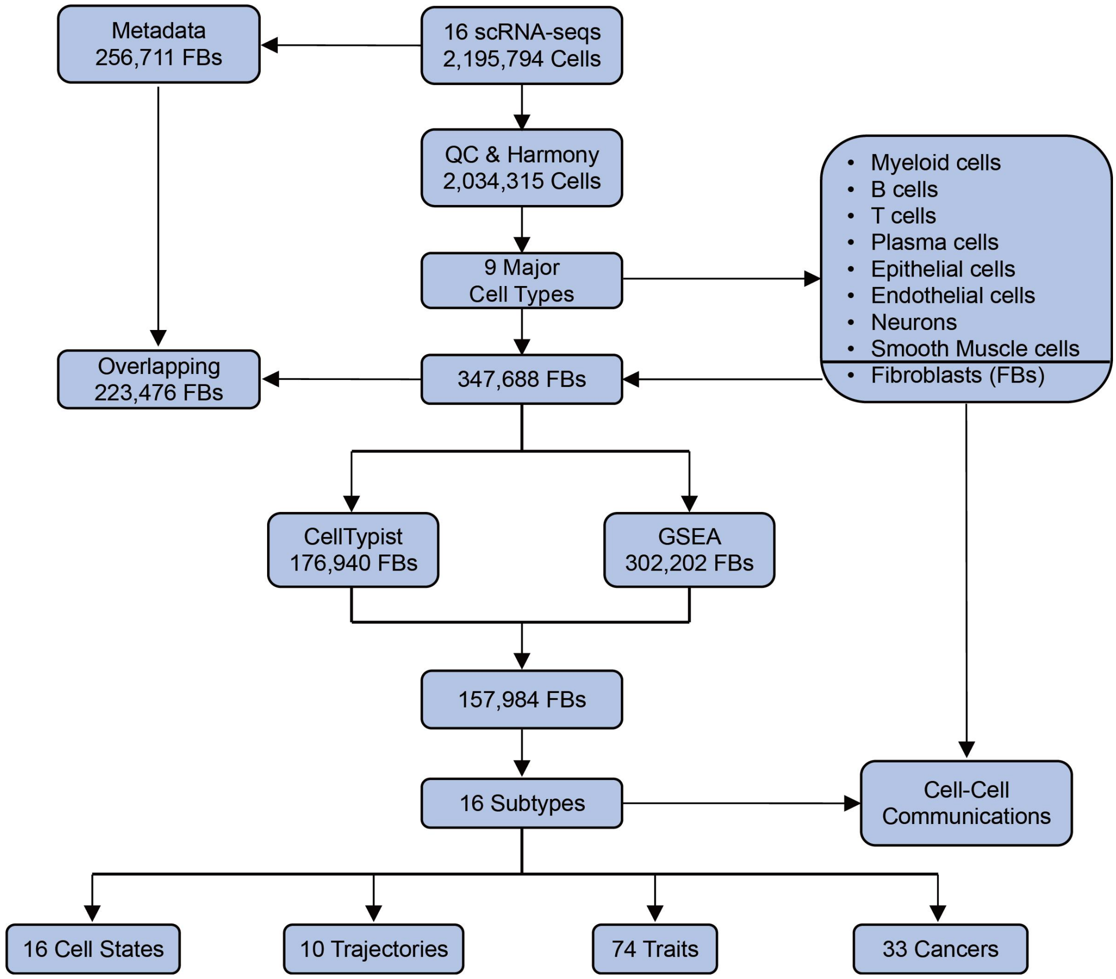

Supplemental Figure 1. The workflow of data processing.

scRNA-seq: single-cell RNA sequencing;

snRNA-seq: single-nucleus RNA sequencing;

FBs: fibroblast cells.

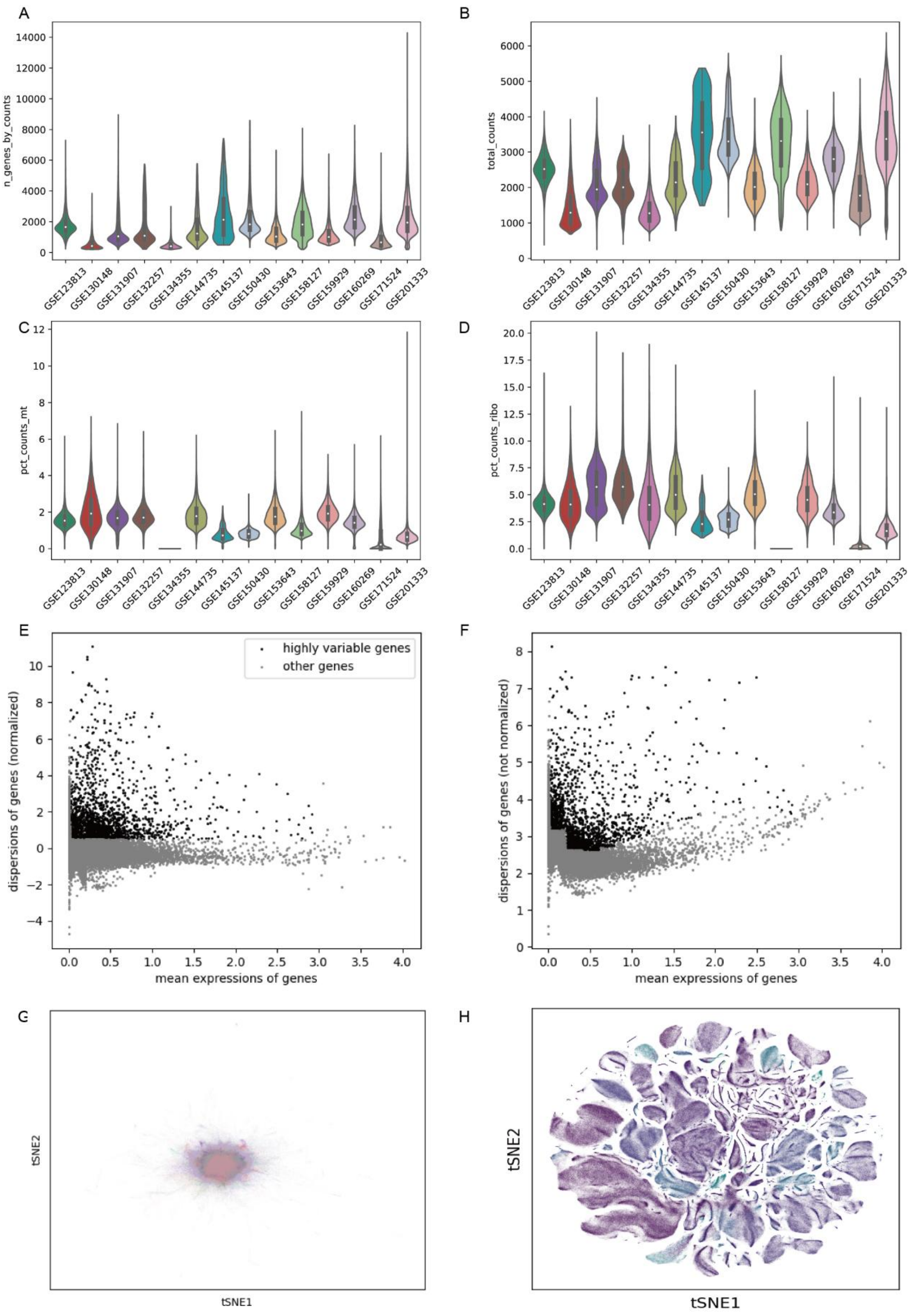

Supplemental Figure 2. The quality control of datasets.

(A-D) Violin plots of gene counts **(A)**, gene expression **(B)**, percent of mitochondria genes **(C)**, and percent of ribosomal protein gene expression ratio **(D)**. **(E-F)** Distributions of the gene expression levels. The black dots represented the top 2000 highly variable genes (HVGs)

and the gray dots represented other genes. **(G-H)** The t-distributed Stochastic Neighbor Embedding (t-SNE) distribution illustration of all cells before batch correction **(G)** and after batch correction **(H)**. Each dot represented a cell, and the color corresponded to the source dataset.

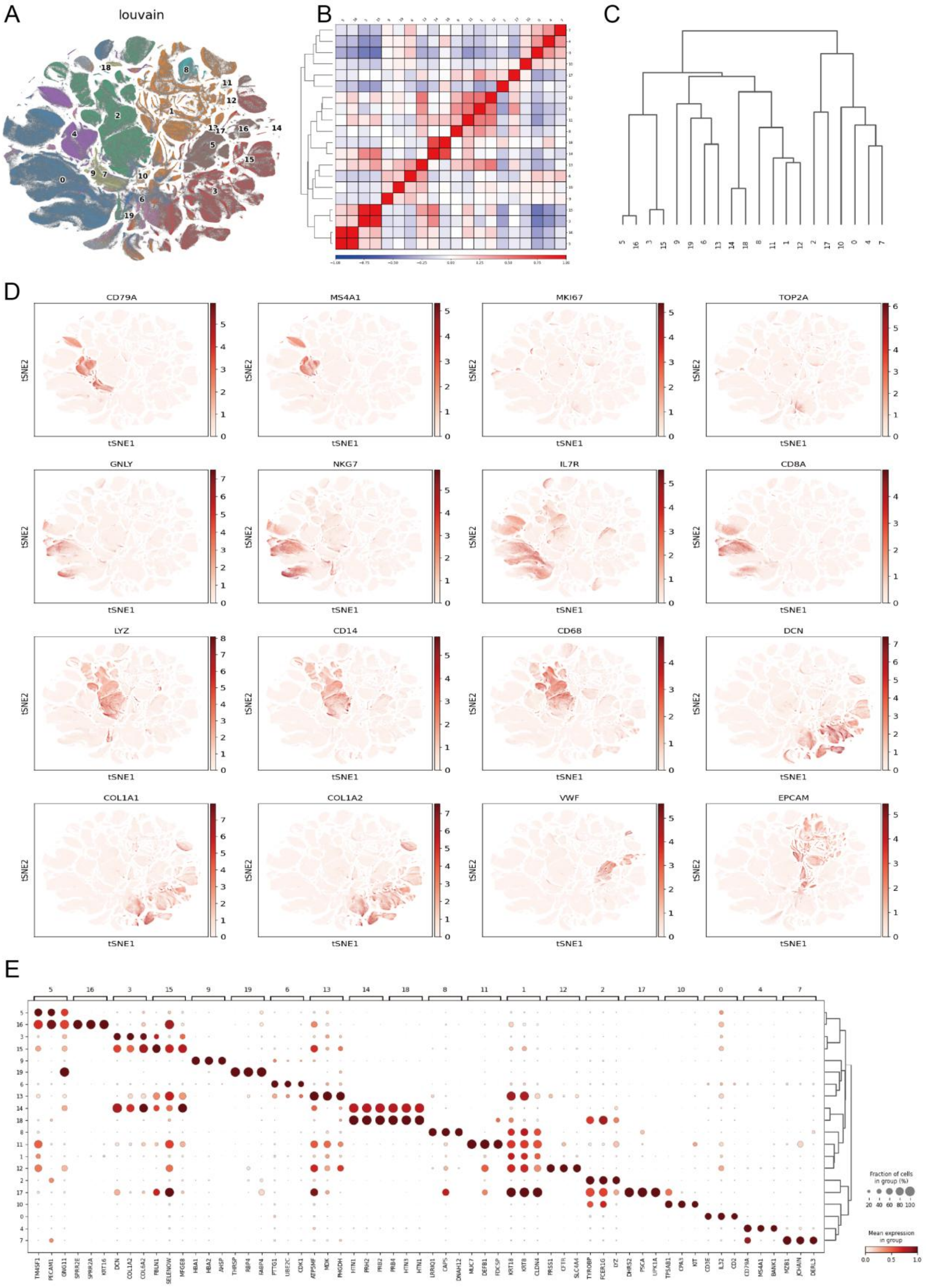

Supplemental Figure 3. Cell clusters and cluster-specific genes of all cells.

(A) The t-SNE visualization of 29 clusters identified by the Louvain method using all cells. **(B)** Associations among the 29 clusters. The color was proportional to the Spearman correlation coefficient (SCC). **(C)** The dendrogram of 29 clusters identified using hierarchical

clustering. **(D)** The t-SNE plot of all cells colored by different marker genes. Each panel represented a single gene colored by the gene expression levels. **(E)** Dot plots of the top 3 differentially expressed genes (DEGs) for each cell cluster selected by log fold change (logFC). The dot size was proportional to the fraction of cells in each group, and the color was proportional to the mean gene expression.

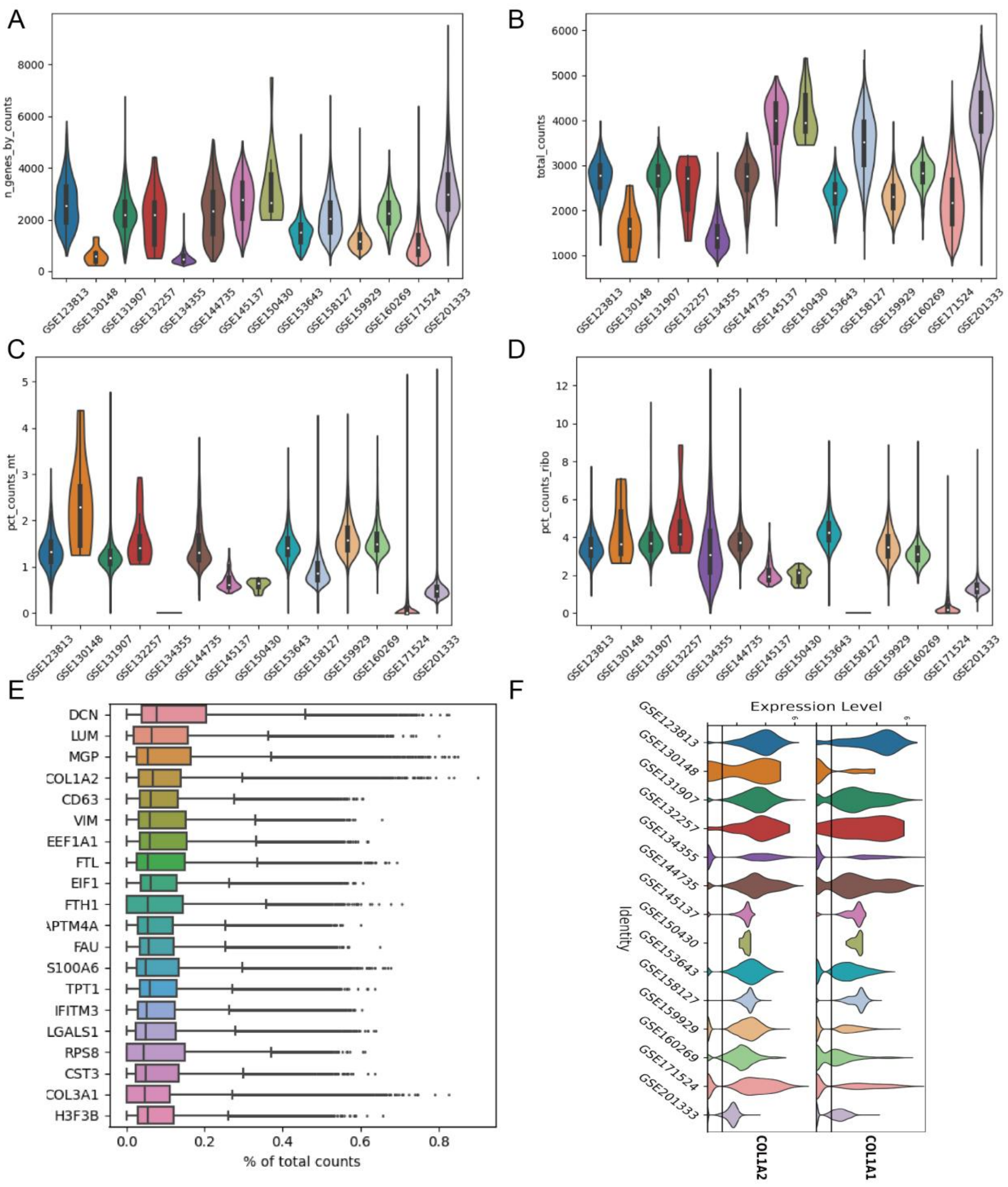

Supplemental Figure 4. The quality control of fibroblast subtypes.

(A-D) Violin plots of gene counts **(A)**, gene expression **(B)**, percent of mitochondria genes **(C)**, and percent of ribosomal protein gene expression ratio **(D)**. **(E)** Boxplots of the top 20 highly expressed genes. The boxes were bounded by the first and third quartiles with a vertical line at the median, and whiskers extend to the maximum and minimum value. **(F)** Violin plots of the two classical fibroblast markers, i.e., *COL1A1* and *COL1A2*, in each dataset.

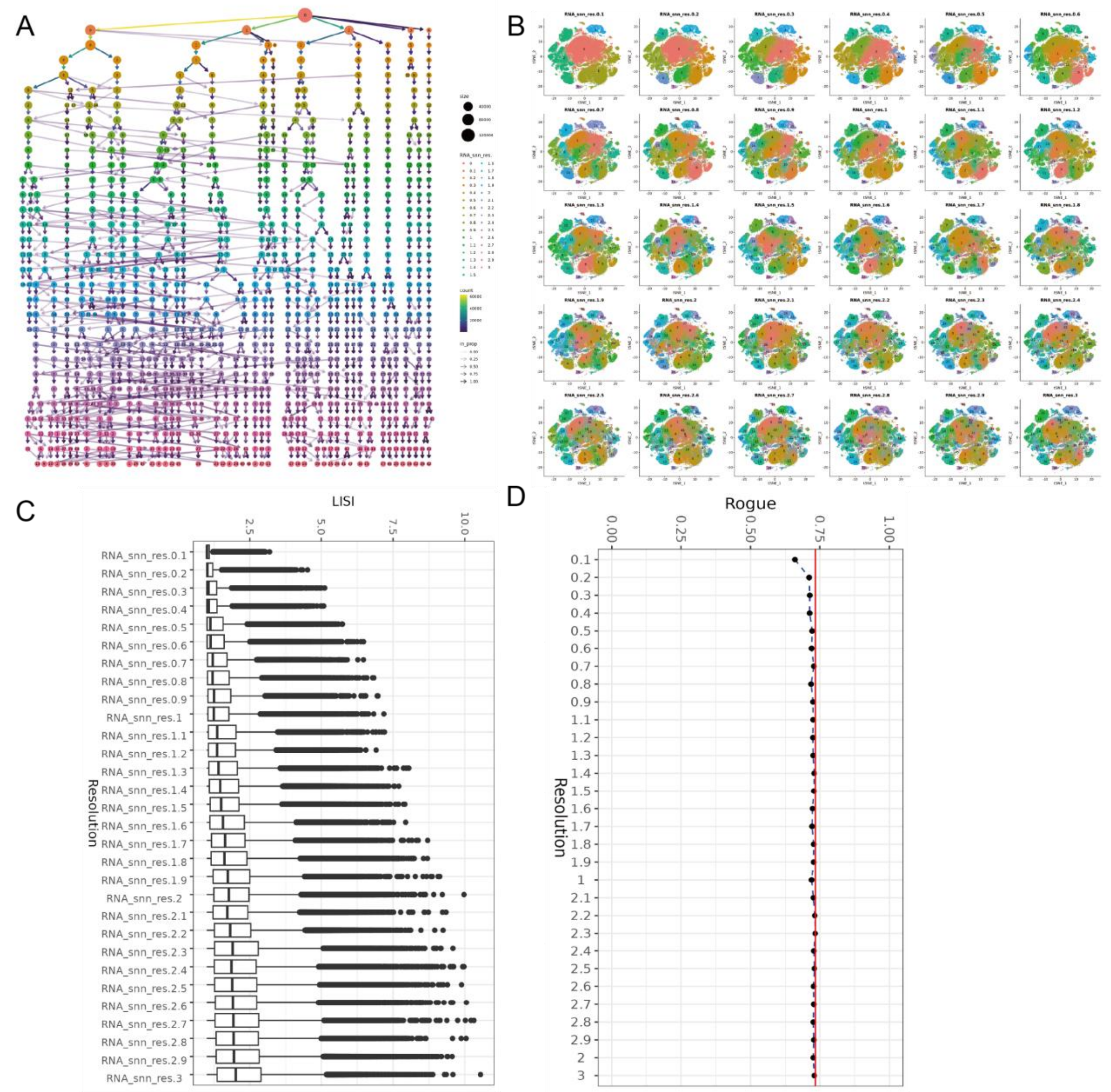

Supplemental Figure 5. The resolution of split clusters and the purity of cell clusters.

(A) The branch tree obtained using different resolutions ranging from 0 to 3. The top branch showed the clusters (first line) obtained using resolution = 0. The bottom branch showed the clusters obtained using resolution = 3. **(B)** The t-SNE distribution of cell clusters obtained using resolution from 0 to 3. **(C)** Boxplots of local inverse Simpson's index (LISI) at different resolution values from 0 to 3. The boxes were bounded by the first and third quartiles with a vertical line at the median, and whiskers extend to the maximum and minimum value. **(D)** Line-point showed the purity value evaluated by the Ratio of Global Unshifted Entropy (ROGUE) algorithm, with the resolution value ranging from 0 to 3. The purity of the cell population was the highest when resolution = 2.3.

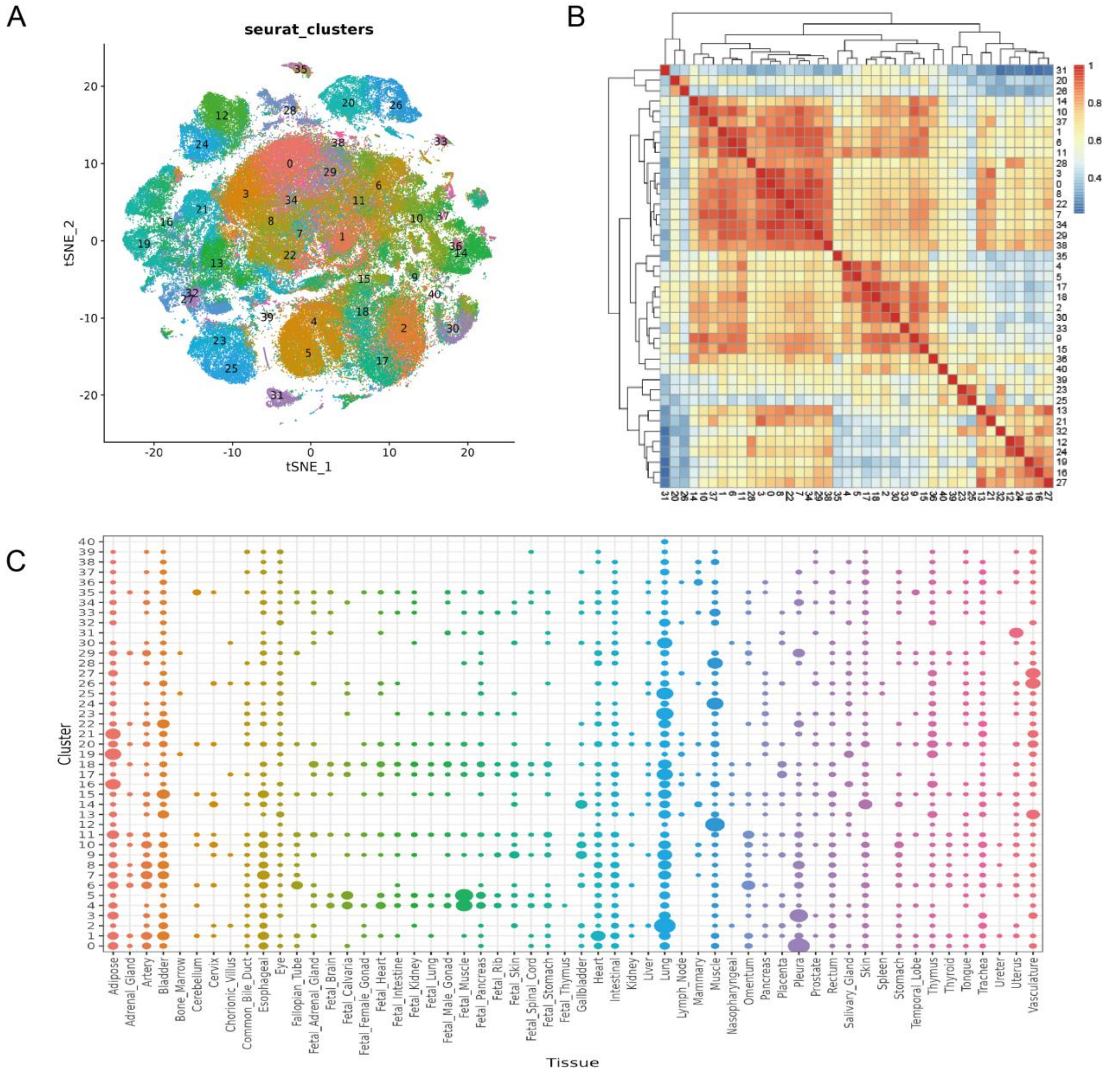

Supplemental Figure 6. The distribution of fibroblast clusters.

(A) The t-SNE plot of cell cluster distribution at resolution = 2.3. **(B)** Heatmap of 26 fibroblast clusters. Color indicates the correlation between two clusters measured by SCC. **(C)** The dot plots of cluster-specific genes. Dot size is proportional to the fraction of cells in a cluster. Dot color is proportional to mean gene expression levels.

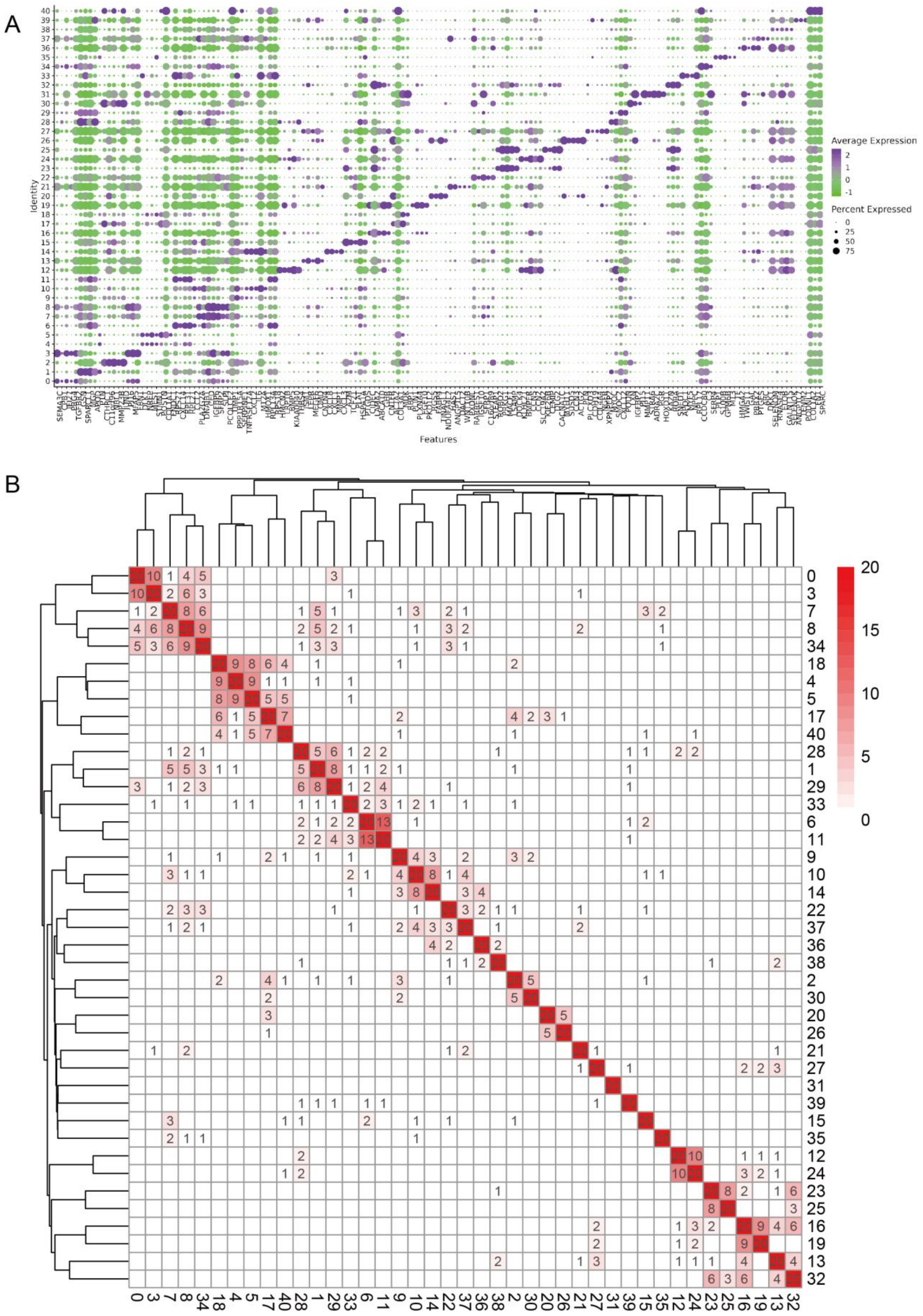

Supplemental Figure 7. The DEGs of fibroblast clusters.

(A) The dot plot showing the top 5 DEGs of each cell cluster (selected based on logFC). Dot size is proportional to the fraction of cells in a cluster. Dot color is proportional to mean gene expression levels. **(B)** Heatmap showing the unsupervised hierarchical clustering of 41

fibroblast clusters using the top 20 DEGs per cluster. Color is proportional to the overlapping genes.

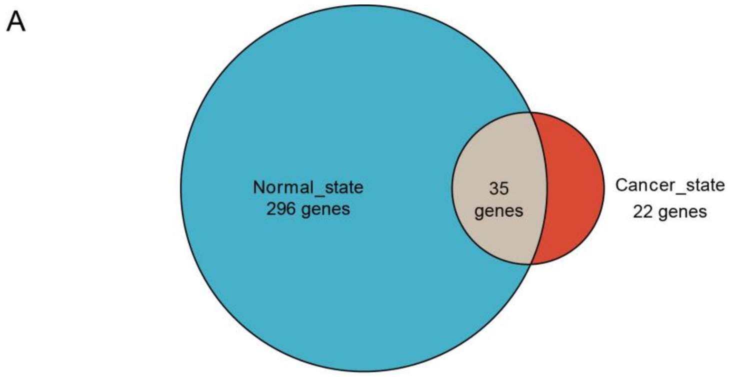

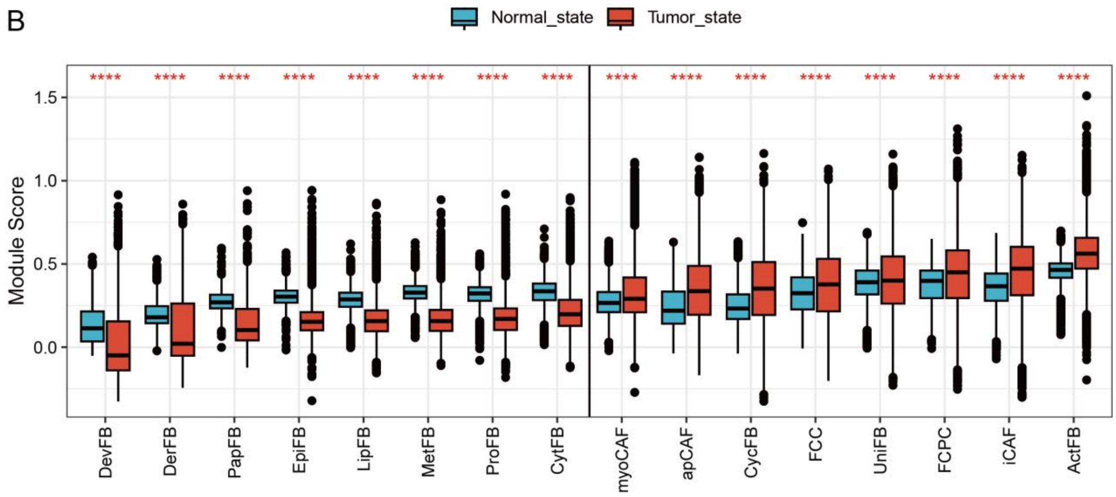

Supplemental Figure 8. The normal and CAF-like state of 16 fibroblast subtypes.

(A) Venn diagram showing the marker genes of the normal and the tumor states. **(B)** Box plots showing the module score for each fibroblast subtype. The boxes were bounded by the first and third quartiles with a horizontal line at the median, and whiskers extend to the maximum and minimum value. A two-sided t-test was used to test the significance of the module score between the normal and the CAF-like state. *: p-value < 0.05 & p-value > 0.01; **: p-value <0.01 & p-value > 0.001; ***: p-value < 0.001 & p-value > 0.0001; ****: p-value < 0.0001.

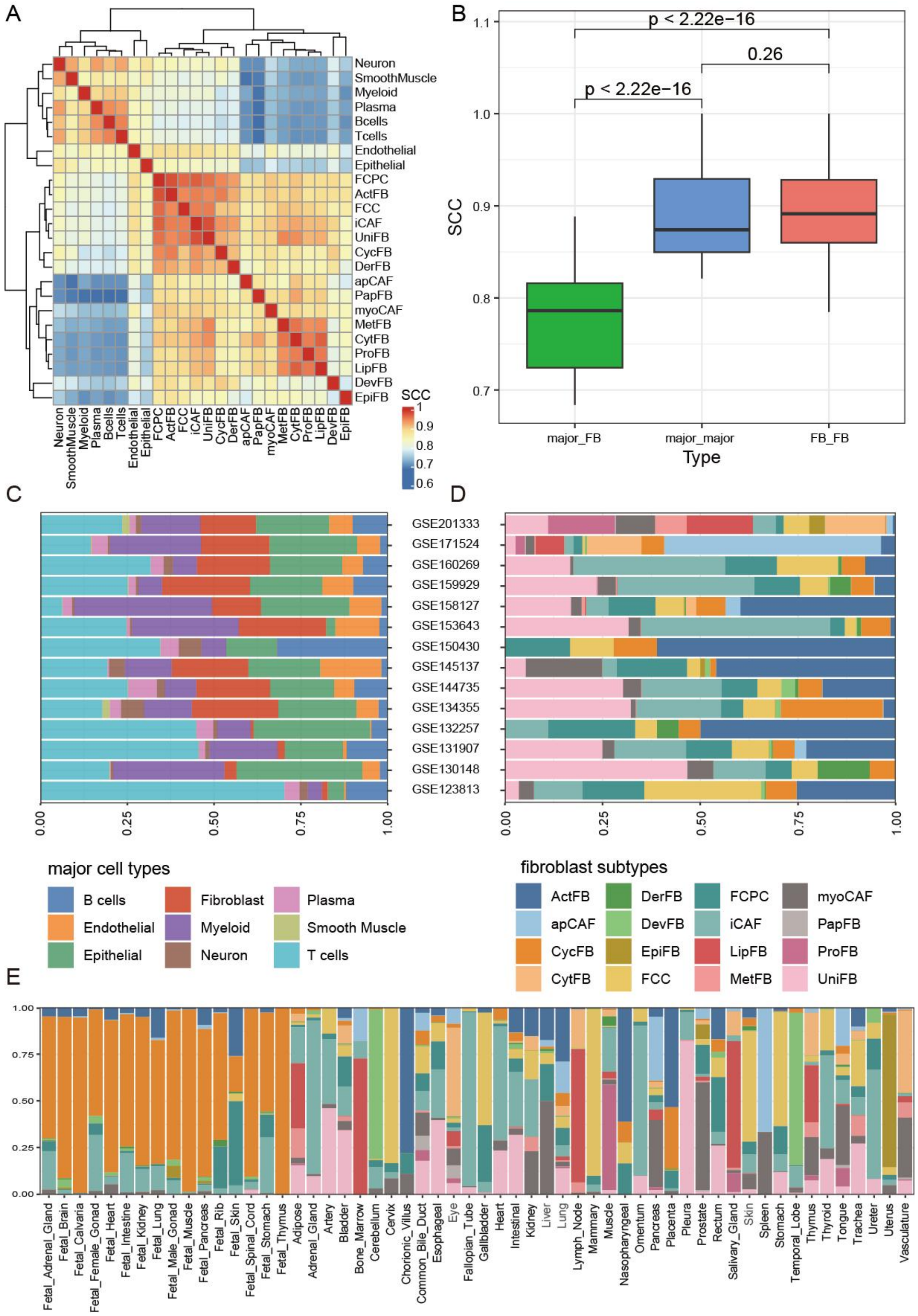

Supplemental Figure 9. Cell component of datasets and tissues.

(A) Heatmap showing the spearman correlation coefficient (SCC) of 8 major cell types and 16 fibroblast subtypes. (B) Boxplot showing the SCC of major_FB (blue box) (among 16

fibroblast subtypes and 8 major cell types), major_major (blue box) (among 8 major cell types), and FB_FB (red box) (among 16 fibroblast subtypes). **(C)** The cell type composition in each dataset. Bar length is proportional to the cell type composition. Bar color corresponds to cell types. **(D)** The fibroblast subtype composition in each dataset. Bar length is proportional to the cell type composition. Bar color corresponds to fibroblast subtypes. **(E)** The proportions of fibroblast subtypes across different tissues. Bar color indicates fibroblast subtypes.

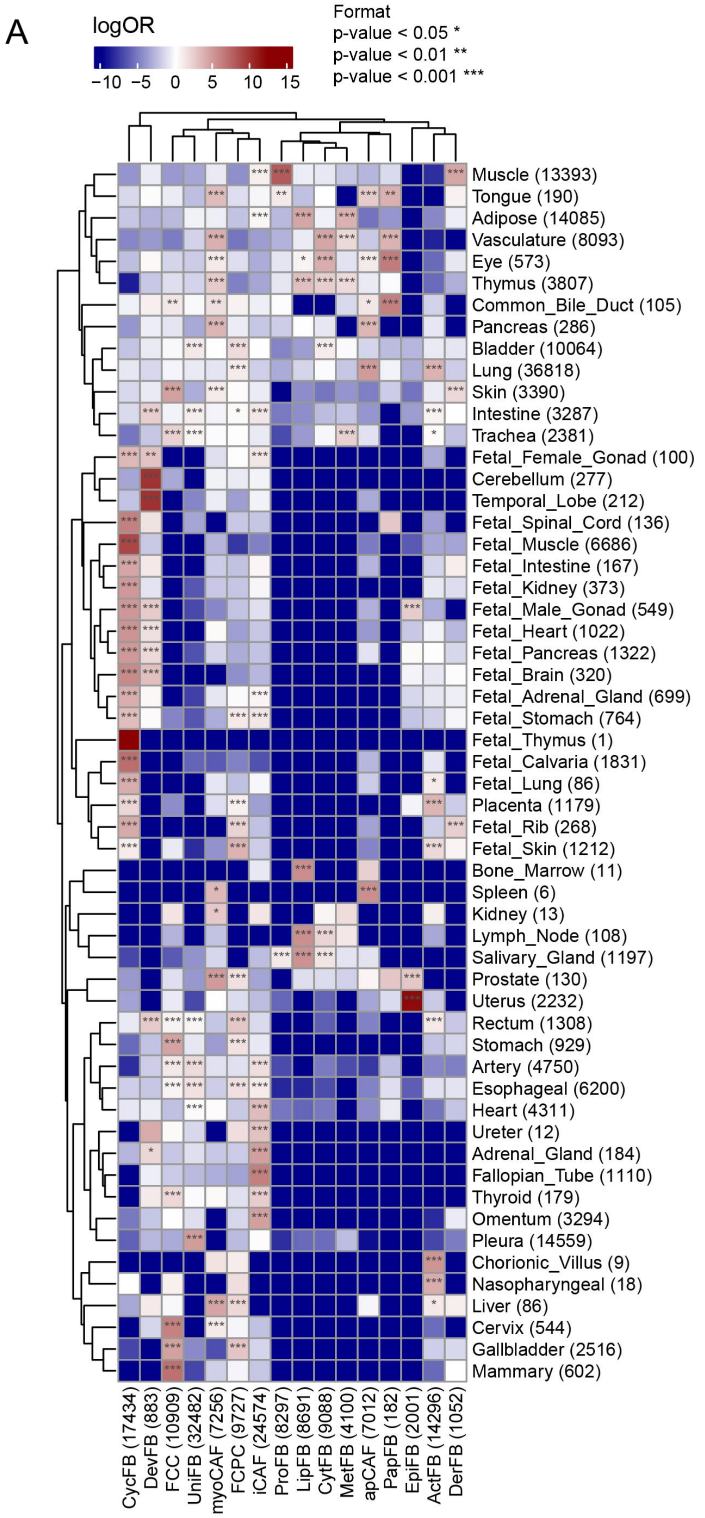

Supplemental Figure 10. The enrichment of 16 fibroblast subtypes across 56 tissues.

Heatmap showing whether the fibroblast subtypes are significantly enriched in different tissues. The p-value and odds ratio (OR) were calculated by Fisher's Exact test. The color is proportional to the log2(OR) (blue indicating log2(OR) < 0 and red indicating log2(OR) > 0). *: p-value < 0.05 & p-value > 0.01; **: p-value <0.01 & p-value > 0.001; ***: p-value <

0.001.

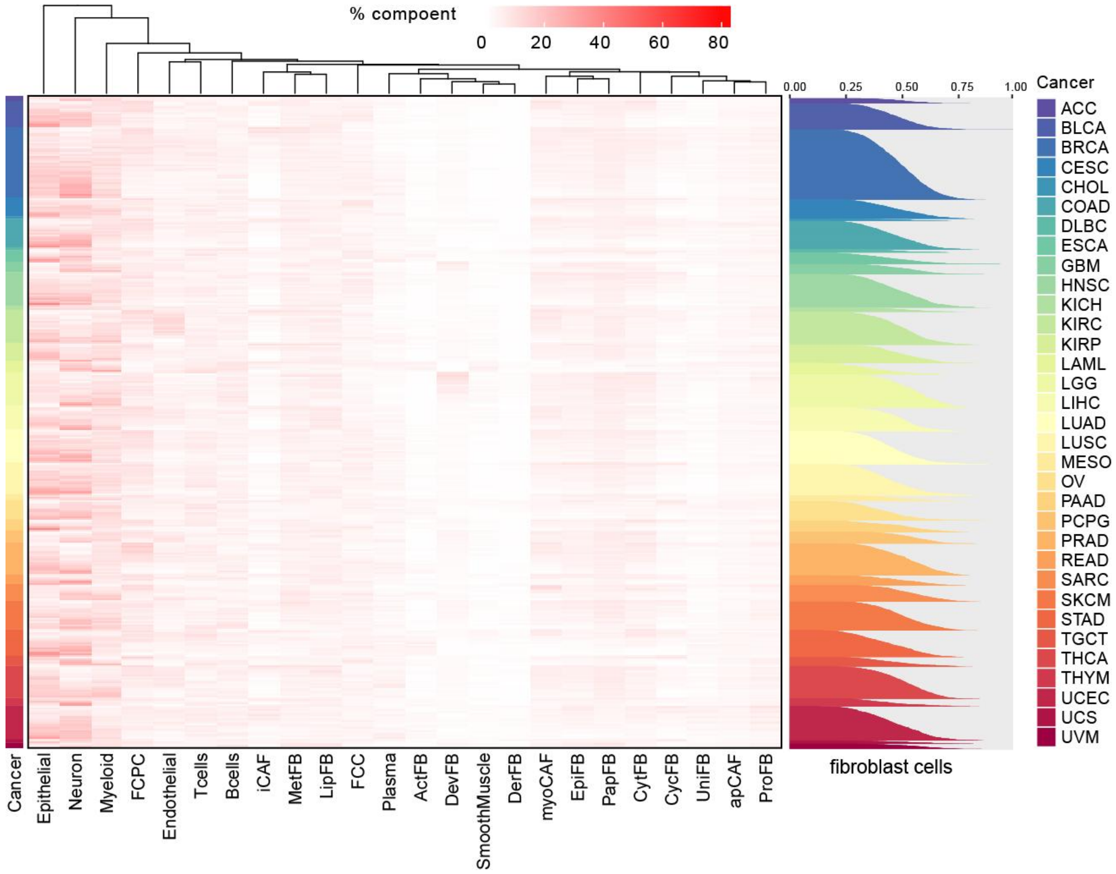

Supplemental Figure 11. Composition of 16 fibroblast subtypes and 8 other major cell types across 33 cancer types in the TCGA dataset.

The x-axis represents the 8 major cell types and 16 fibroblast subtypes. The y-axis corresponds to the TCGA sample. Bar height indicates the overall proportion of each fibroblast subtype across the samples.

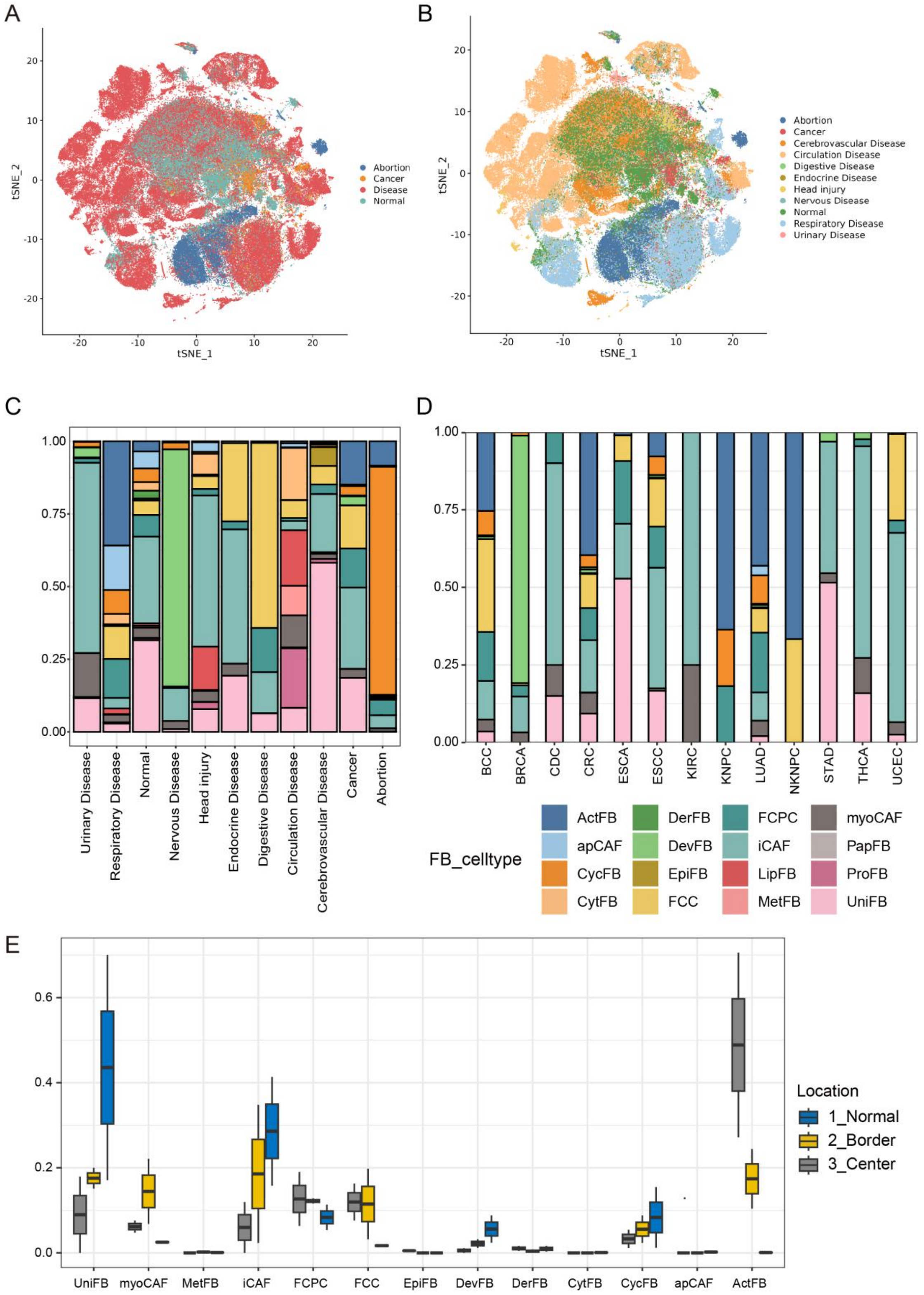

Supplemental Figure 12. Clinical characterization of fibroblast subtypes.

(A) The t-SNE plot colored by tissue classification. **(B)** The t-SNE plot colored by ICD-11 classifications. **(C)** Composition of fibroblast subtypes in different diseases. Bar color indicates fibroblast subtypes. **(D)** Composition of fibroblast subtypes in 12 cancer types. **(E)**

Boxplots showing the proportion of fibroblast subtypes in different tumor samples. The boxes were bounded by the first and third quartiles with a horizontal line at the median, and whiskers extend to the maximum and minimum value.

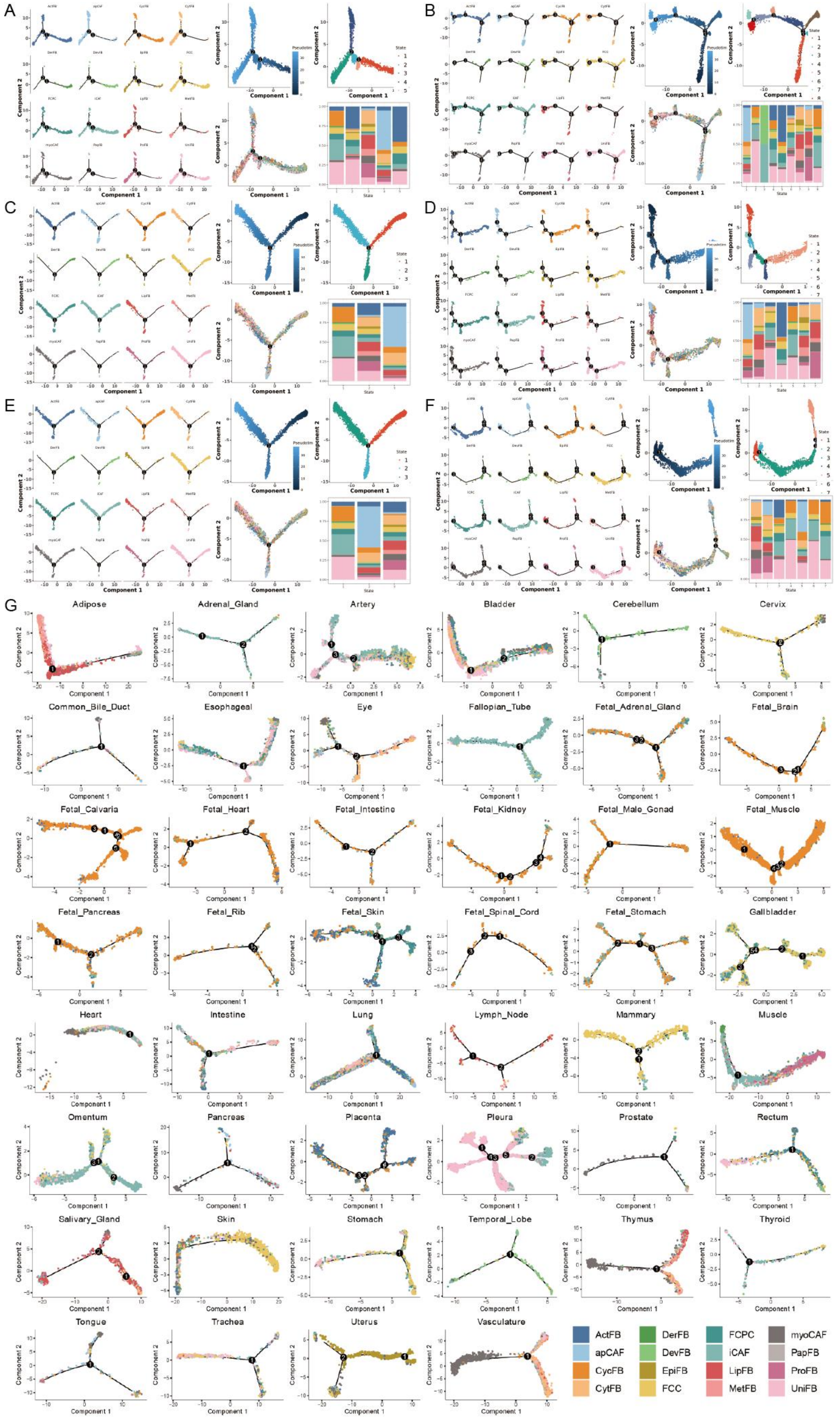
A
B
C
D
E
F
G
Component 1
Component 2
Pseudotime
State
Adipose
Adrenal_Gland
Artery
Bladder
Cerebellum
Cervix
Common_Bile_Duct
Esophageal
Eye
Fallopian_Tube
Fetal_Adrenal_Gland
Fetal_Brain
Fetal_Calvaria
Fetal_Heart
Fetal_Intestine
Fetal_Kidney
Fetal_Male_Gonad
Fetal_Muscle
Fetal_Pancreas
Fetal_Rib
Fetal_Skin
Fetal_Spinal_Cord
Fetal_Stomach
Gallbladder
Heart
Intestine
Lung
Lymph_Node
Mammary
Muscle
Omentum
Pancreas
Placenta
Pleura
Prostate
Rectum
Salivary_Gland
Skin
Stomach
Temporal_Lobe
Thymus
Thyroid
Tongue
Trachea
Uterus
Vasculature
ActFB
apCAF
CycFB
CytFB
DerFB
DevFB
EpiFB
FCC
FCPC
iCAF
LipFB
MetFB
myoCAF
PapFB
ProFB
UniFB

Supplemental Figure 13. Trajectory analyses.

(A-F) Trajectory analyses using monocles six times with down-sampling. (**G**) Trajectory analyses using monocles across 46 tissues.

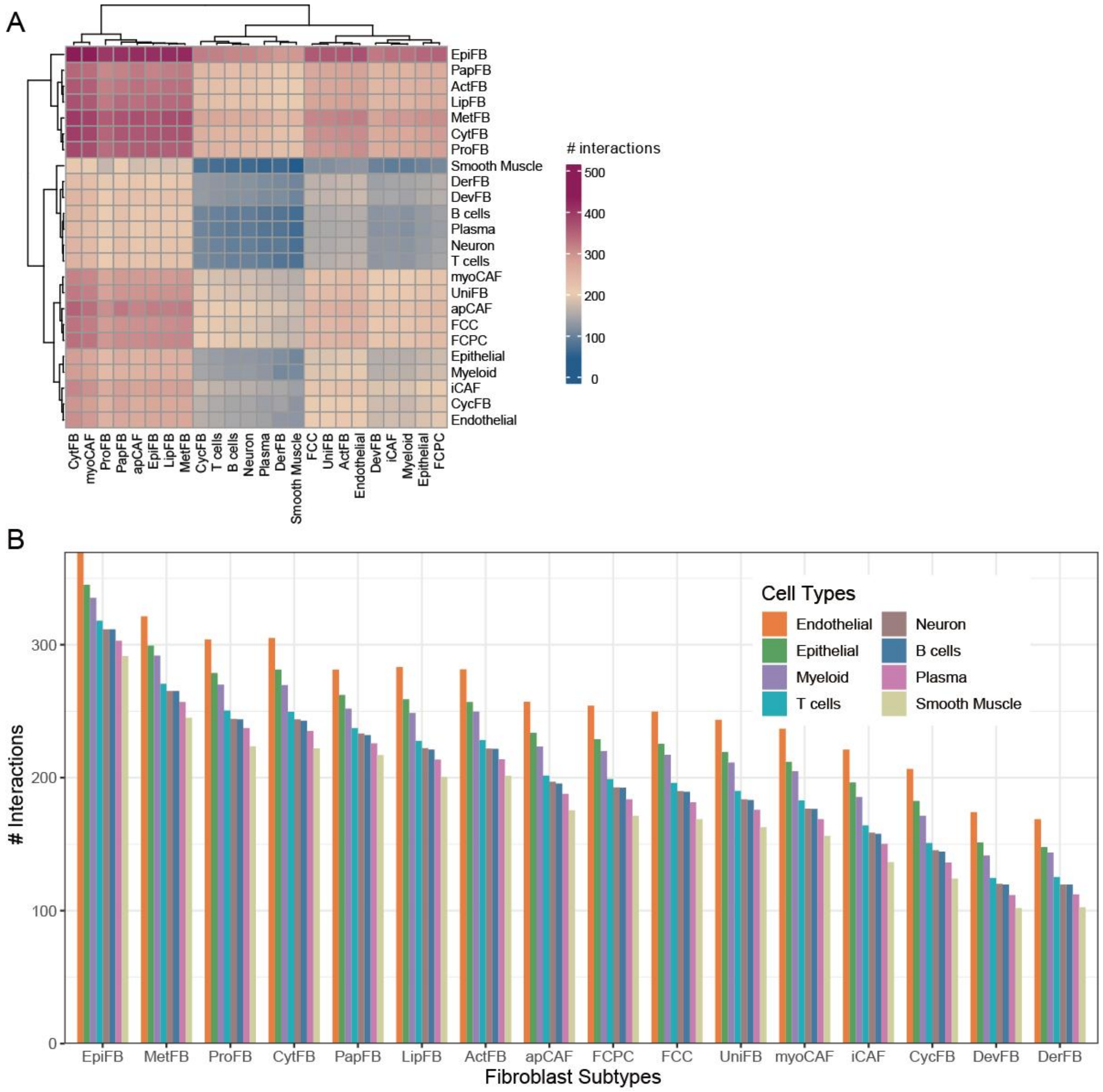

Supplemental Figure 14. Cell communications of fibroblast subtypes and other cell types.

(A) The heatmap showing the total number of ligand-receptor interactions between cell types obtained using CellPhoneDB. The color represents the number of ligand-receptor interactions.

(B) Bar plots showing the interactions between fibroblast subtypes and non-fibroblast cell types.

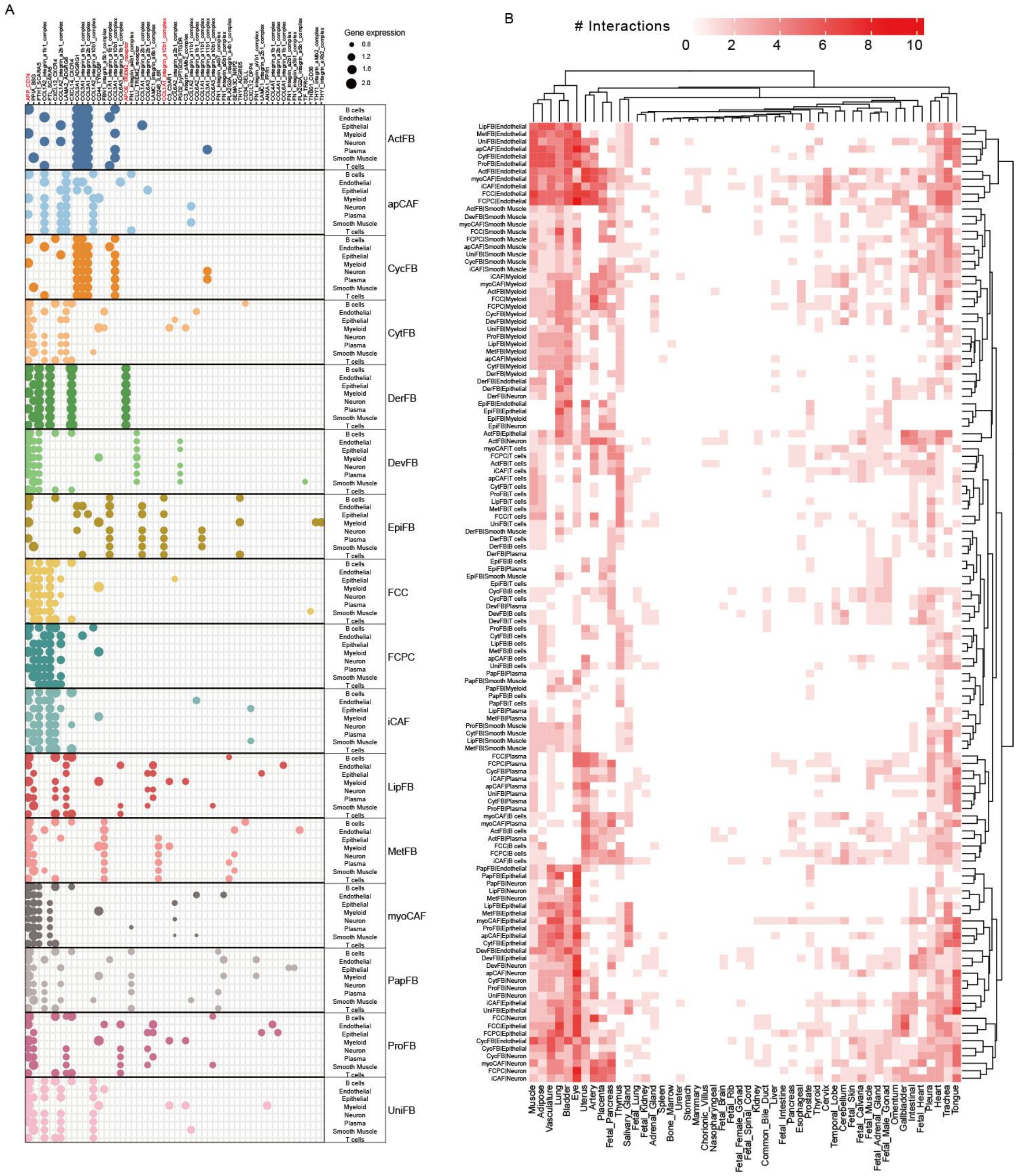

Supplemental Figure 15. Cell-cell communications between 16 fibroblast subtypes and 8 other major cell types.

(A) The dot plot showing the top five ligand-receptor interactions identified by using CellPhoneDB. Dot size is proportional to the average expression of the interacting ligand and receptor from the two cell types, where the first molecule (before the underline) was from the fibroblast subtypes (e.g., *APP* in ActFB) and the second molecule was from those non-fibroblast cell types (e.g., *CD74* in B cells). Three pairs were highlighted in red to show that *APP_CD74* was identified in all pairs of cell types, *COL1A1_integrin_a10b1_complex*

was only identified in EpiFB, and *APOE_TREM2_receptor* was unique to DerFB. (**B**) The number of cell-cell communications between 16 fibroblast subtypes and 8 other major cell types across 50 tissues.

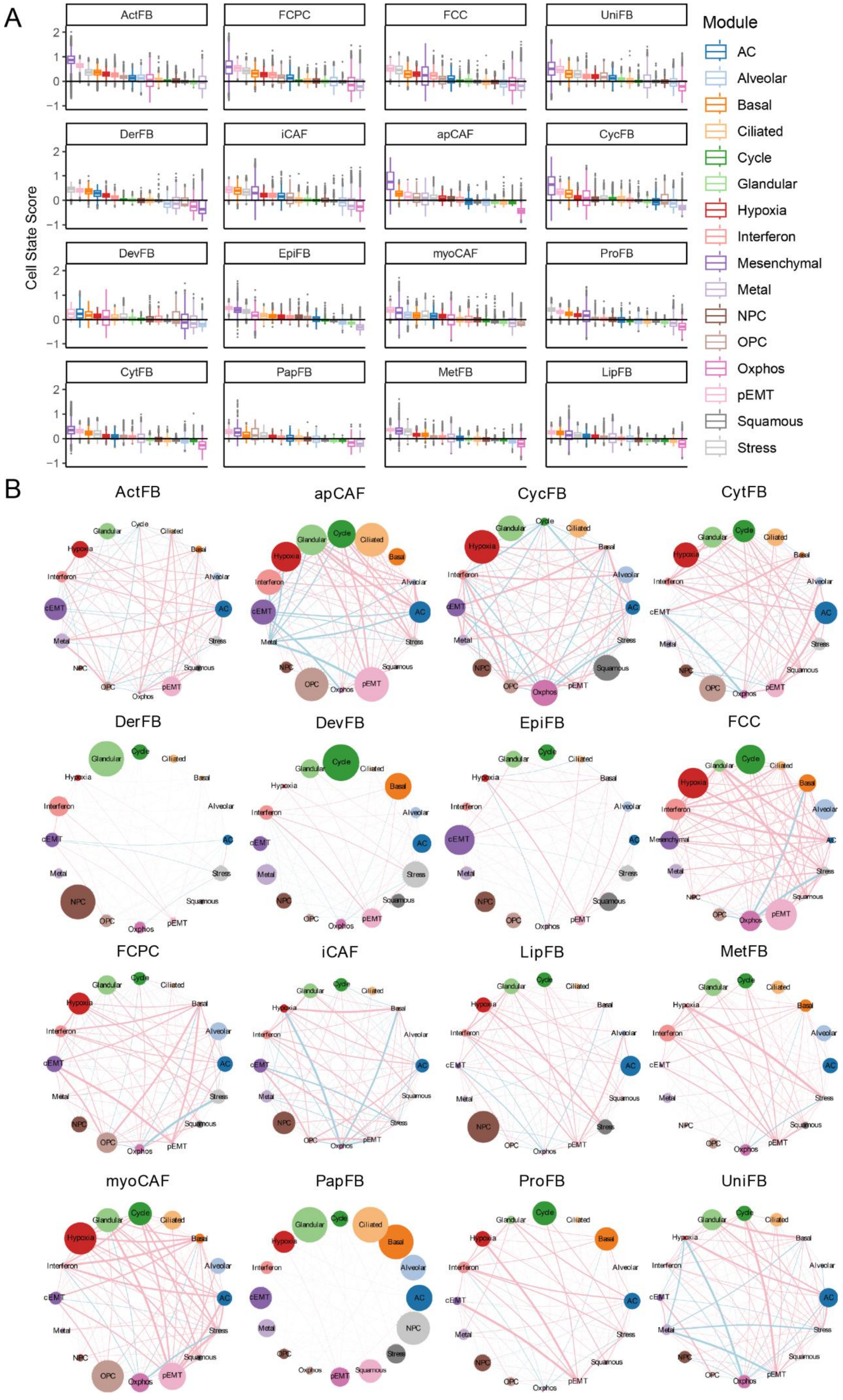
A
ActFB
FCPC
FCC
UniFB
DerFB
iCAF
apCAF
CycFB
DevFB
EpiFB
myoCAF
ProFB
CytFB
PapFB
MetFB
LipFB
Cell State Score
2
1
0
−1
Module
AC
Alveolar
Basal
Ciliated
Cycle
Glandular
Hypoxia
Interferon
Mesenchymal
Metal
NPC
OPC
Oxphos
pEMT
Squamous
Stress
B
ActFB
apCAF
CycFB
CytFB
DerFB
DevFB
EpiFB
FCC
FCPC
iCAF
LipFB
MetFB
myoCAF
PapFB
ProFB
UniFB
Cycle
Ciliated
Basal
Alveolar
AC
Stress
Squamous
pEMT
Oxphos
OPC
NPC
Metal
cEMT
Interferon
Hypoxia
Glandular
Mesenchymal

Supplemental Figure 16. Cell state of fibroblast subtypes.

(A) Box plots of the enrichment score for 16 fibroblast subtypes colored by 16 cell states. The boxes were bounded by the first and third quartiles with a horizontal line at the median, and whiskers extend to the maximum and minimum value. **(B)** The interaction of cell state in each fibroblast subpopulation. In each plot, dot size is proportional to the module expression, and line width is proportional to the SCC between the two states. Line color indicates the direction of SCC, with red for positive correlation and blue for negative correlation.

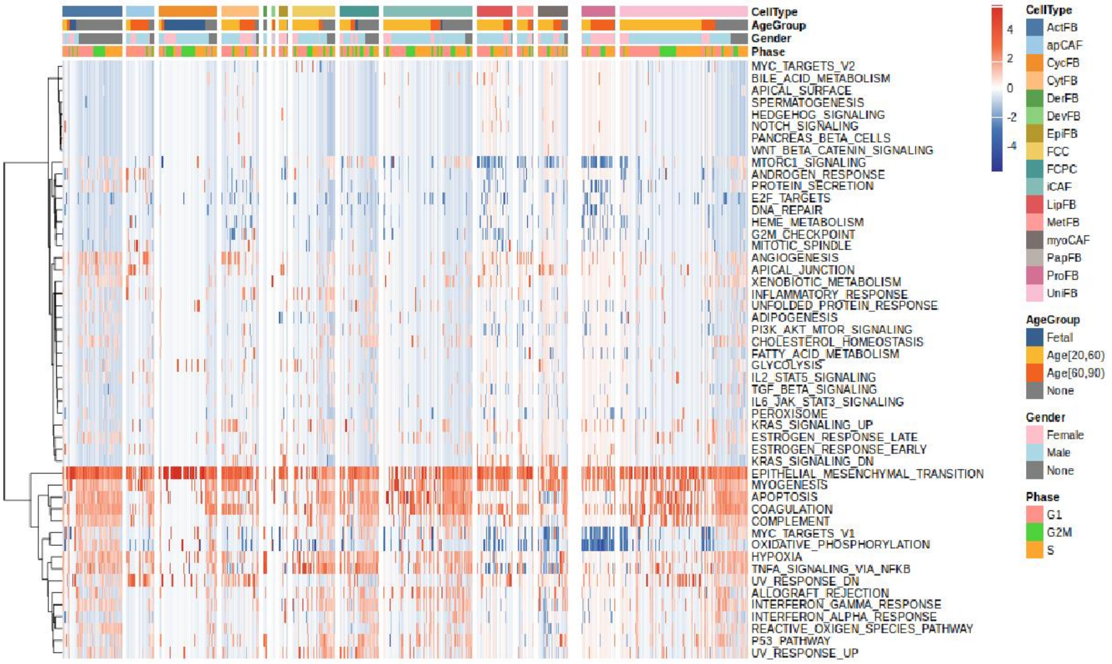

Supplemental Figure 17. GSEA analysis of fibroblast subtypes using the HALLMARK gene sets from MSigDB.

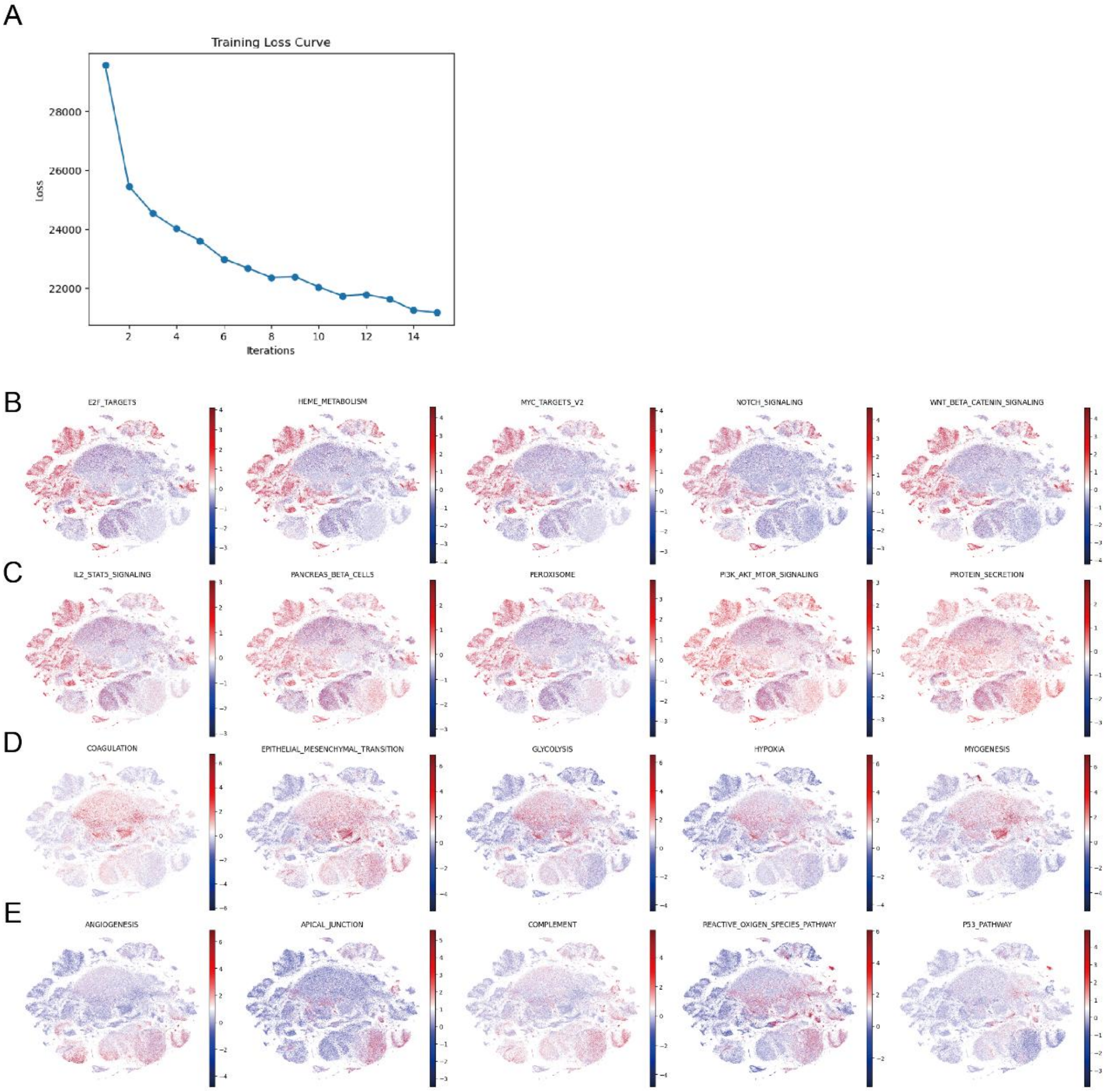

Supplemental Figure 18. Pathway activity scores by VEGA.

(A) The curve of loss by the variational autoencoders enhanced by gene annotations (VEGA).

(B-E) The t-SNE plots for representative hallmark pathways. Color is proportional to the score calculated by VEGA.

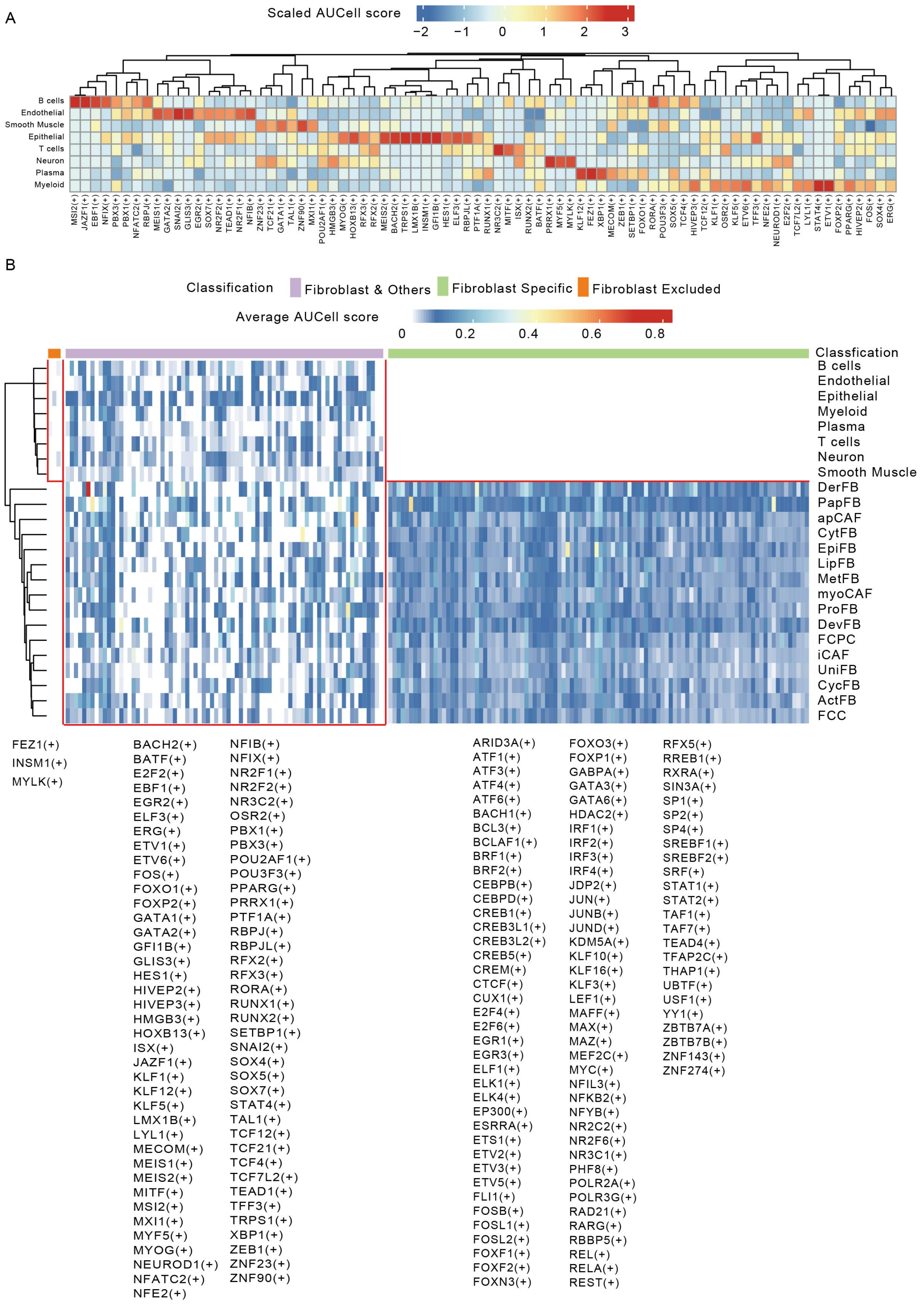

Supplemental Figure 19. The transcription factor of 8 major cell types and 16 fibroblast subtypes.

(A) Transcription factors (TFs) of B cells, endothelial, smooth muscle, epithelial, T cells,

neurons, plasma, and myeloid. (**B**) The cell type-specific TFs of 8 other major cell types and 16 fibroblast subtypes, including 3 TFs in non-fibroblast cells, 107 fibroblast-specific TFs, and 77 shared TFs in both fibroblast subtypes and major cell types.

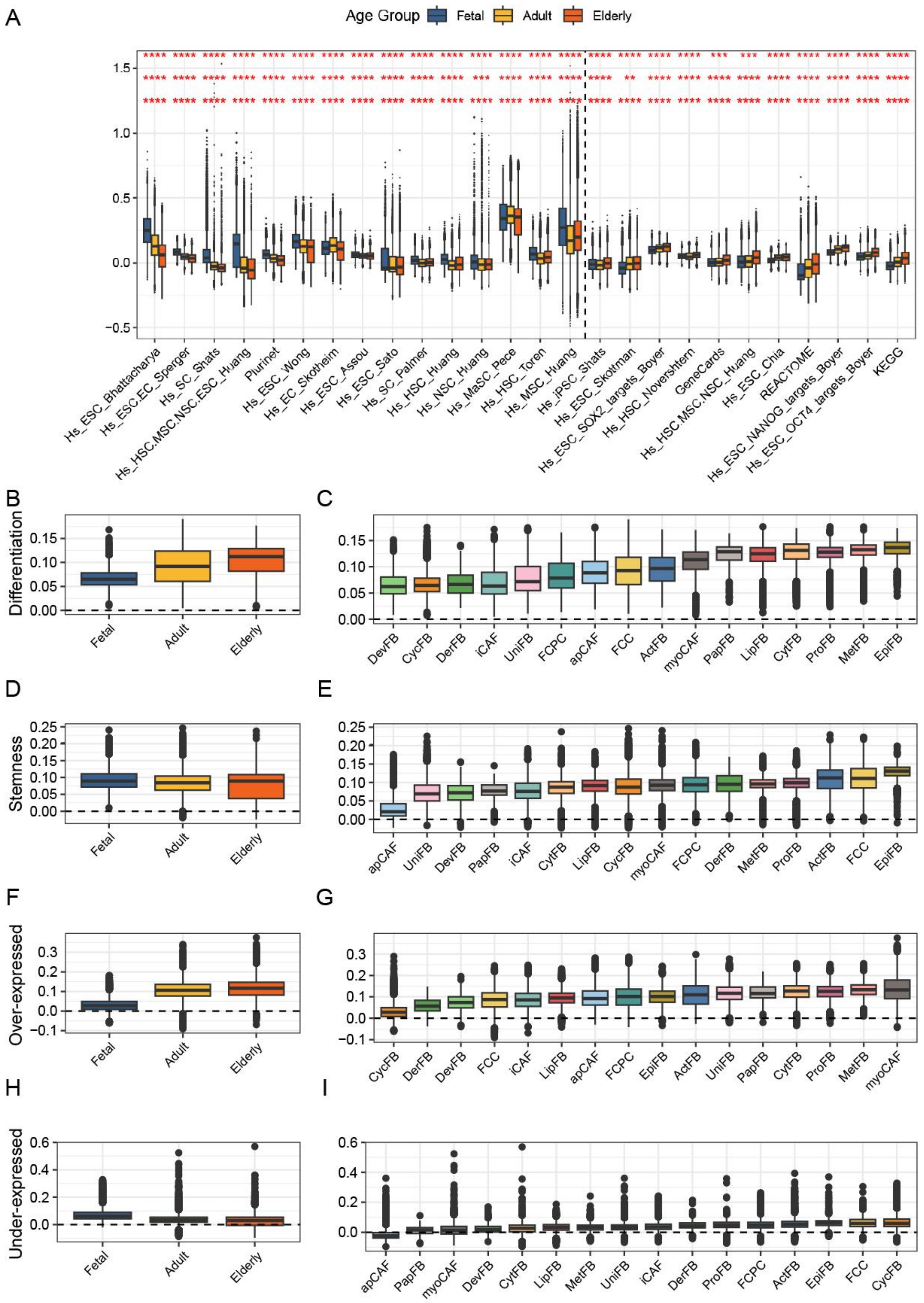

Supplemental Figure 20. Age-related marker score of fibroblast subtypes.

(A) Box plots showing the module score for each age group. The boxes were bounded by the first and third quartiles with a horizontal line at the median, and whiskers extend to the maximum and minimum value. A two-sided Wilcoxon test was used to test the significance of

expression level between fetal and adult (the first line of significance), adult and elderly (the second line of significance), and fetal and elderly (the third line of significance). *: p-value < 0.05 & p-value > 0.01; **: p-value <0.01 & p-value > 0.001; ***: p-value < 0.001. **(B-C)** Box plots showing the differentiation score by age groups (**B**) or by fibroblast subtypes (**C**). **(D-E)** Box plots showing the stemness score grouped by age groups (**D**) or by fibroblast subtypes (**E**). **(F-G)** Box plots showing the over-expressed score grouped by age (**F**) or by fibroblast subtypes **(G)**. **(H)** Box plots showing the under-expressed score grouped by age (**H**) or by fibroblast subtypes **(I)**.

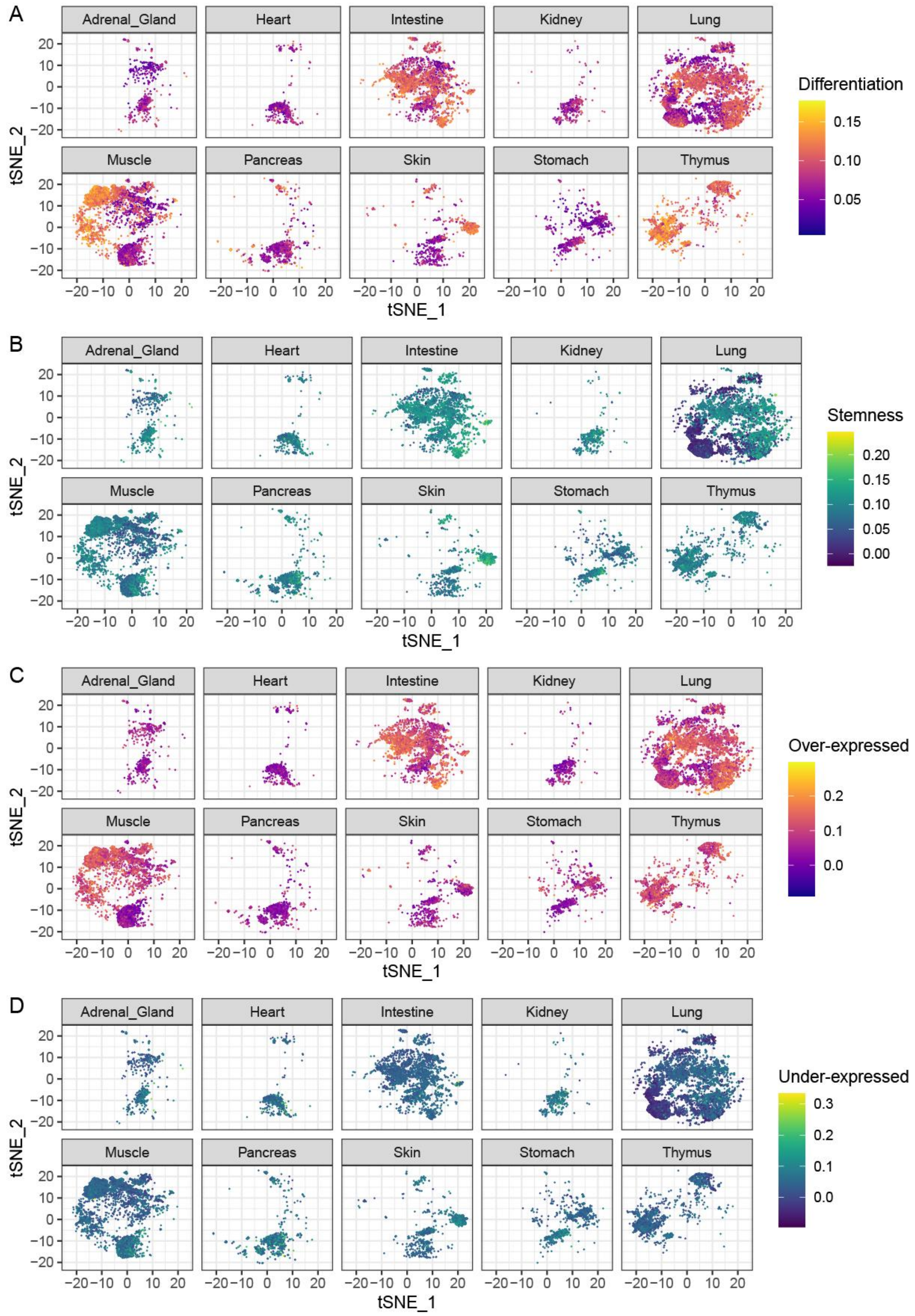

Supplemental Figure 21. Distribution of senescence and stemness scores of fibroblast cells across 10 matched tissues.

(A) The t-SNE plot of the differentiation score. **(B)** The t-SNE plot of the stemness score. (**C**) The t-SNE plot of the over-expressed score. (**D**) The t-SNE plot of the under-expressed score.

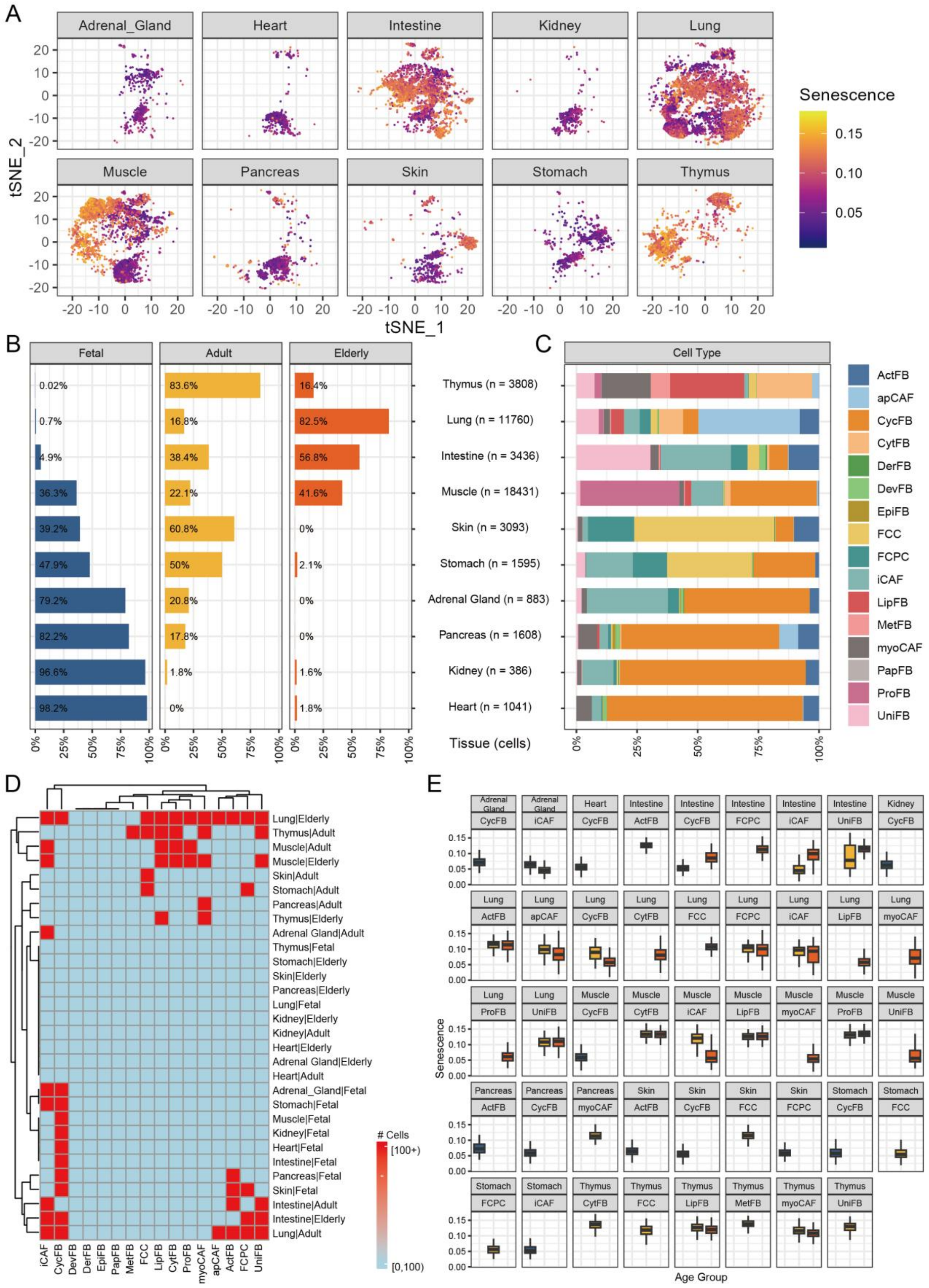

Supplemental Figure 22. Senescence score of fibroblast cells across 10 matched tissues. **(A)** The t-SNE plot of the senescence score. **(B)** Bar plot showing the percentage of fibroblast cells from the fetal, adult, and aged groups. (**C**) Bar plot showing the percentage of fibroblast subtypes across 10 tissues. (**D**) Heatmap showing the number of cells from different fibroblast

subtypes, stratified by the fetal, adult, and aged groups. Red: the condition had more than 100 cells. Blue: the condition had fewer than 100 cells. **(E)** Boxplot of senescence score of fibroblast subtypes across 10 tissues in conditions with at least 100 cells.

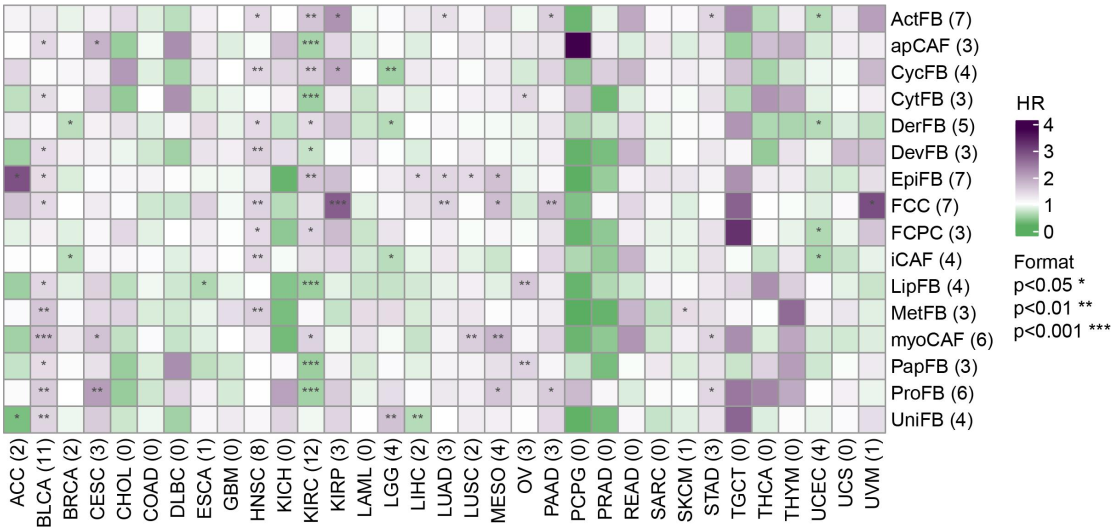

Supplemental Figure 23. Results of the survival analyses using 16 fibroblast subtypes and the TCGA data.

The p-value and hazard ratio (HR) were calculated by log-rank survival analysis. The color is proportional to the log10(HR) (green indicating log10(HR) < 0 and purple indicating log10(HR) > 0). *: p-value < 0.05 & p-value > 0.01; **: p-value <0.01 & p-value > 0.001; ***: p-value < 0.001.

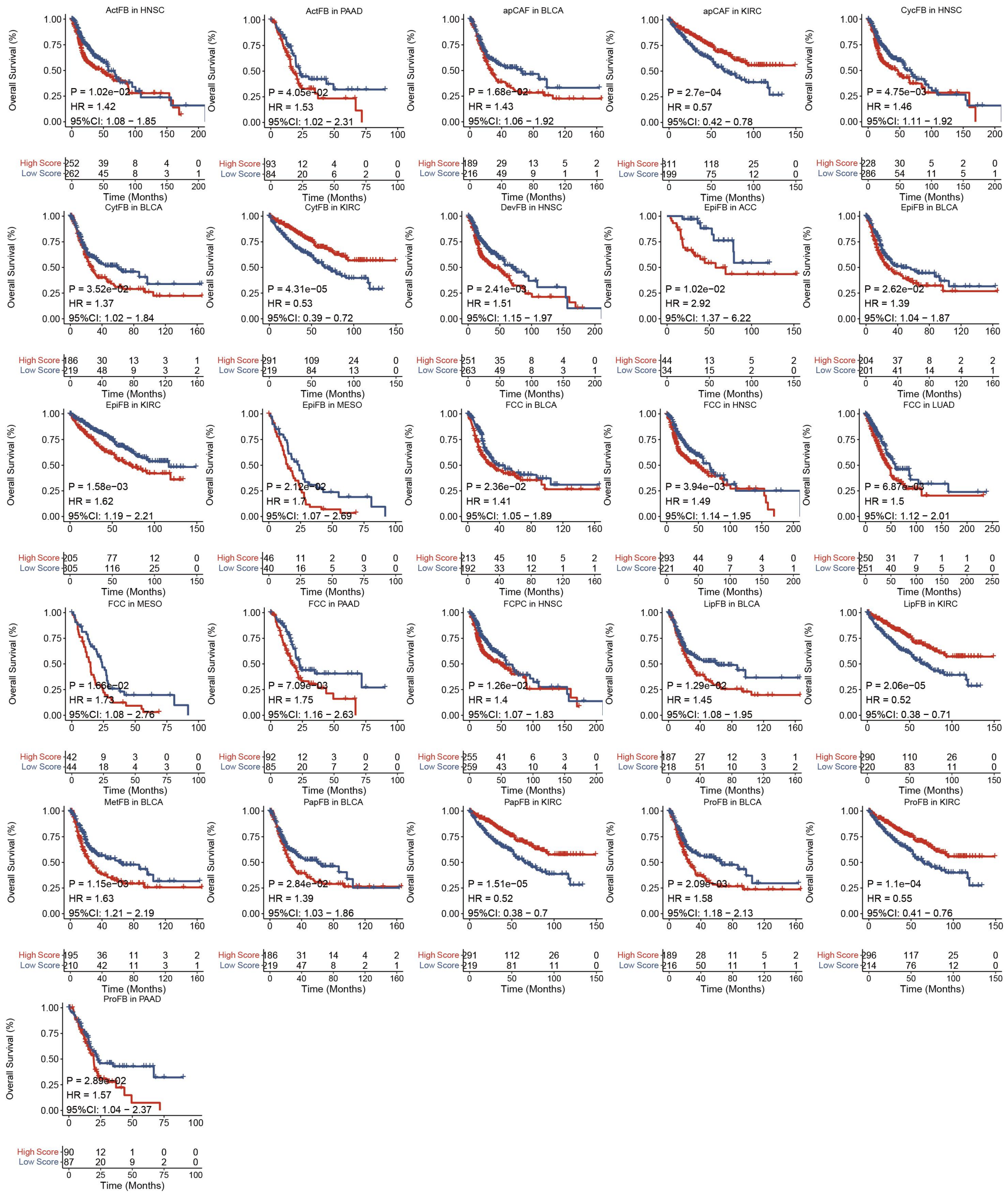

Supplemental Figure 24. The survival plots of samples stratified by the enrichment score across fibroblast subtypes.

In each case, samples were divided into two groups by the mean enrichment score (red: high score group; blue: low score group). The p-value, HR, and 95% CI were calculated by log-rank survival analysis. The full names of cancer types were available in **Table S8.** The statistical values of log-rank and Cox survival analysis are in **Table S14.** Only the conditions that were significant by both the Cox analysis and the log-rank survival analysis were shown.
